# Size-Dependent Oxidation of Copper Nanostructured Electrocatalysts Produced by Spark Ablation

**DOI:** 10.1021/acselectrochem.6c00062

**Published:** 2026-05-18

**Authors:** Johannes Sterzinger, Bogdan Gulie, Vincenz Maier, Tim Steeger, Carsten Peters, Nikolaos Patelis, Elena Gubanova, Marc Willinger, Aliaksandr S. Bandarenka

**Affiliations:** a Physics of Energy Conversion and Storage, 9184 Technical University of Munich,James-Franck-Str. 1, 85748 Garching, Germany; b Chair of Electron Microscopy, Technical University of Munich, Chemistry Department, Lichtenbergstraße 4, 85748 Garching, Germany; c Catalysis Research Center, TUM, Ernst-Otto-Fischer-Str. 1, 85748 Garching, Germany

**Keywords:** Copper nanoparticles, electrochemical oxidation, size-dependent oxidation, spark ablation, nanoparticle synthesis

## Abstract

Nanostructured materials play a crucial role in today’s society and are widely used across a range of fields, from catalysis to energy storage systems. However, their synthesis and characterization are often time-, cost-, and energy-consuming. This study aims to develop relatively simple approaches for upscaling size-selected nanoparticle synthesis via a plasma-based physical strategy and for determining particle sizes using electrochemical methods. We present a scalable, cost-effective spark ablation synthesis strategy that enables the production of high-purity, non-agglomerated copper (Cu) nanoparticles, directly immobilized on a carbon support, without the use of reducing agents or surfactants. This approach achieves high collection efficiency and imposes no limitations on attainable mass loading. To address challenges in nanoparticle size characterization, we introduce a simple electrochemical approach, namely anodic potential sweep oxidation, for determining particle size based on the size-dependent oxidation potential. For the produced Cu nanoparticles, a strong linear correlation (*R* ^2^ = 0.99) was observed between particle radius and oxidation peak potential. This study demonstrates that the size of approximately spherical Cu nanoparticles can be estimated by simple, cost-effective electrochemical measurements, without the need for complex microscopy techniques. That opens a perspective for the express-assessment of particle size changes during various physicochemical processes, e.g., catalytic reactions.

## Graphical abstract

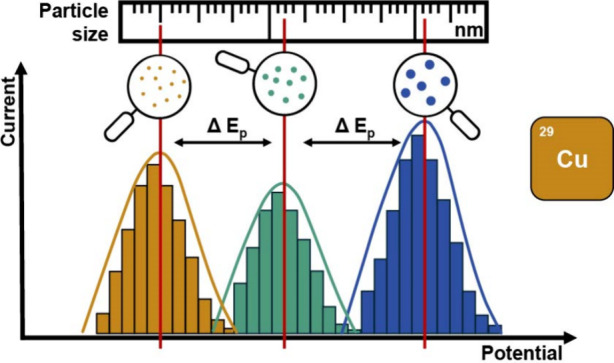

## Introduction

Nanoparticles (NPs) have become integral to a broad spectrum of technological and scientific applications, including electro-optics, sensing, catalysis, energy storage, biomedical technologies, and nanoelectronics.^1–4^ Critical challenges in nanoparticle research include determining and controlling particle size and shape, and ensuring their stability against oxidation. Among metallic nanoparticles, copper (Cu) has emerged as a particularly promising candidate for various catalytic applications due to its natural abundance and cost-efficiency.^5,6^ These advantageous properties position Cu NPs as economically viable alternatives to other noble metal catalysts (such as Pt and Ru) in various electrocatalytic processes, including methanol oxidation, and CO_2_ reduction to produce fuels, fertilizers, and other valuable chemicals.^5,7,8^

While nanoparticles, including Cu NPs, can be synthesized through various methodologies, wet-chemical approaches remain the most prevalent.^9,10^ However, most conventional methods are significantly constrained by the rapid oxidation of Cu upon atmospheric exposure.^11^ Spark ablation presents a compelling alternative by generating nanoparticles within an inert gas environment, thereby eliminating the oxidative degradation and contamination issues inherent to wet-chemical synthesis.^12,13^ This technique produces highly pure metallic nanoparticles free of surface oxidation and chemical residues, owing to the absence of chemical reagents and reactions.^14,15^ Furthermore, spark ablation offers environmental advantages by eliminating the need for chemical precursors and additives while producing no hazardous waste. Combined with relatively high energy efficiency, these benefits establish spark ablation as a simple, scalable, and cost-effective nanoparticle production method.^16^

Typically, characterization of NPs requires measurements using microscopic techniques such as scanning electron microscopy (SEM) and transmission electron microscopy (TEM) to determine particle morphology and verify size distributions. However, such a characterization often requires sophisticated and expensive instrumentation. Consequently, simpler and more cost-effective alternative methods would be highly desirable for routine, rapid NP size assessment. While experimental and theoretical research suggest a correlation between the electrode oxidation potential of metal nanoparticles and their size, direct electrochemical measurements that systematically examine this effect remain scarce.^17–19^ Theoretical predictions indicate that the oxidation potential increases with increasing nanoparticle size.^18^ However, certain microscopy studies have reported contradictory findings, claiming that small copper clusters on gold substrates exhibit enhanced stability against electrochemical oxidation compared to their bulk counterparts.^20,21^

Size-dependent electrochemical behavior of metal nanoparticles has been already investigated in several systems. In particular, voltammetric studies on Au,^22–26^ Ag,^1^ and Pd^27^ nanoparticles by Zamborini, Compton, Brainina, Buttry and co-workers have demonstrated that nanoparticle size can influence oxidation peak potentials and related electrochemical characteristics. Similar effects have also been reported for Cu nanoparticles,^28^ although these systems remain comparatively less explored. Most of these previous studies have focused on fundamental electrochemical behavior, while systematic investigations of supported Cu nanoparticle ensembles and their potential use for electrochemical size analysis under well-defined conditions are still limited.

This work presents an optimized approach for size-selected nanoparticle production coupled with a proof-of-concept demonstration of electrochemical nanoparticle size assessment via anodic oxidation of Cu NPs in acidic media. We produce Cu NPs of different sizes in the range of ca. 2 nm to 20 nm using spark ablation and correlate the anodic oxidation potential with the average Cu NP size to establish an analytical methodology for express-assessment of the average nanoparticle size, particularly for catalytic applications, where agglomeration, dissolution, and reconstruction can take place during the process.

## Experimental Section

### Production of Cu NPs via Spark Ablation

For the synthesis of Cu NPs, a commercial spark ablation device (VSP-G1, VSPARTICLE B.V., Delft, The Netherlands) was employed (Supporting Information, Figures S1−S7). In the process, argon gas (99.999%, Westfalen AG) served as the inert carrier gas, while bulk copper electrodes (99.99% purity, 27 mm length, 6 mm diameter, VSPARTICLE B.V.) were used as the source material. As depicted in Figure 1a, an electric spark is initiated when primary electrons, accelerated by a high voltage, collide with and ionize argon atoms, generating a plasma between the electrodes.^29,30^

**Figure 1:**
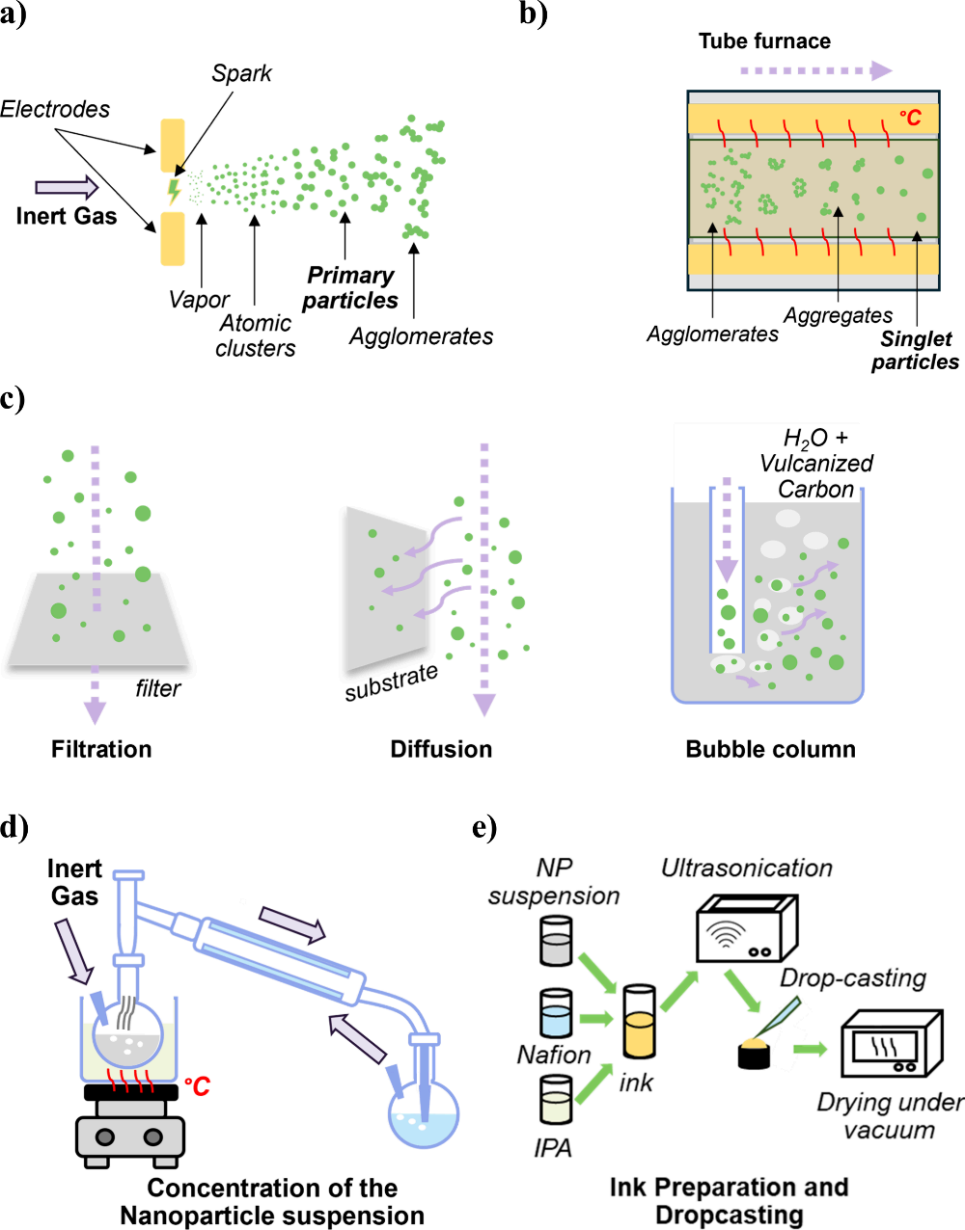
Overview of the different process steps of the nanoparticle synthesis. All steps are performed under an inert gas atmosphere: (a) process of spark ablation, (b) in-flight annealing of nanoparticles in a tube furnace, (c) different methods for the immobilization of the nanoparticles: filtration, diffusion, bubble column with a Vulcan carbon (VC) suspension. (d) Setup used for concentrating the nanoparticle (Cu/VC) suspension under an argon atmosphere. (e) Ink preparation and drop casting of Cu/VC catalyst onto a GC electrode.

During the spark discharge, the intense localized heating partially vaporizes the Cu electrodes. The resulting Cu vapor is rapidly cooled by the flowing argon gas, leading to the nucleation and formation of Cu NPs. By adjusting the carrier gas flow rate and the applied voltage, nanoparticles with varying sizes can be synthesized (Supporting Information, Figure S7).

At carrier gas flow rates exceeding approximately 5 L/min, predominantly primary nanoparticles with approximately spherical shape are formed.^30^ In contrast, lower flow rates tend to result in agglomerated structures, so-called agglomerates, with irregular morphologies. These agglomerates can be transformed into more uniform, spherical single particles through in-flight annealing in a tube furnace downstream of the spark generator.^31^ (Supporting Information, Figure S7). For this purpose, a R50/500/12 tube furnace (Nabertherm, Germany) with a glass tube having an inner diameter of 1.8 cm and a 42 cm length of constant heat was employed. This annealing process involves the partial or complete melting of particles, followed by recrystallization, with the resulting particle size and morphology being influenced by both the furnace temperature and the carrier gas flow rate.^32^ Typically, an increase in the size of the primary particles and a decrease in the agglomerates can be expected, as the large fractal-like agglomeration structures get transformed into smaller spherical particles. However, far exceeding the size of the small primary particles they consist of.^31,32^ The process of in-flight annealing is illustrated in Figure 1b. For our experiment, a temperature of 500 °C, around 50% of the melting point of bulk copper, was selected.

Nanoparticle immobilization from the carrier gas flow was carried out using three different methods (filtration, diffusion, bubble column approach), as illustrated in Figure 1c. The first two techniques utilize modules provided by VSPARTICLE. Employing the filtration method, the substrate functions as a physical filter, directly capturing nanoparticles. Conductive substrates enable the direct utilization of the substrate-nanoparticle assembly as a working electrode in an electrochemical setup. Alternatively, if the substrate is non-conductive, the particles can be extracted from the filter and processed into nanoparticle ink. The diffusion method relies on the Brownian motion of nanoparticles, enabling their deposition onto a substrate positioned parallel to the particle stream. The third technique, based on the utilization of a bubble column, was adapted specifically for our advanced setup based on other reported approaches in the literature.^33^ In this method, nanoparticles are immobilized in a liquid medium via gas bubbling, enabling their collection and stabilization in a suspension.

To maximize nanoparticle collection efficiency in the bubble column, the liquid level, *h*, should be as high as possible. (Supporting Information, Figure S9) The column was filled with 50-150 mL of liquid. Further, 8 mg of pretreated Vulcan carbon (VC) were added to the solution. The deposition time was set to approximately 5 h, yielding a Cu loading of approximately 1% on carbon, as determined by our TGA measurements (Supporting Information, Figure S10).

After deposition, the solution was ultrasonicated in the bubble column for no more than 10 min to minimize nanoparticle loss. It was then poured into a beaker and evaporated at 110 °C under an inert gas atmosphere until the volume was reduced to approximately 5 mL of concentrated solution. This approach was chosen because it minimizes losses, especially compared to first filtering the solution, making powder, and then turning it back into ink. This step was not intended to induce particle growth or restructuring, and at this temperature, significant ripening of Cu nanoparticles on VC was not observed (Supporting Information, Figure S12). After evaporation, the solution was ultrasonicated for 10 min to ensure proper dispersion of the Cu NPs.

The final setup comprises a tube furnace for in-flight annealing, followed by an interchangeable diffusion module for the selective collection of small nanoparticle fractions for TEM analysis. Alternatively, the diffusion module can be replaced by a filter module to remove larger particles from the aerosol stream. However, it is important to note that the optimal deposition times for diffusion-based TEM sampling and for the bubble column may differ. Therefore, implementing a separate, switchable line for TEM grid preparation could be advantageous for future setups. Eventually, the nanoparticles are collected in the bubble column. Similar to previously reported erosion-based setups, the introduction of Vulcan carbon (VC) into the liquid enables the direct synthesis of carbon-supported Cu NPs.^34^

The bubble column exhibits high collection efficiency and, most importantly, imposes no limitations on achievable mass loading. In contrast to direct deposition onto conductive substrates within filter modules, where the deposited mass is inherently constrained, this setup offers scalable flexibility. Parameters such as VC concentration, column liquid volume, or Cu loading can be adjusted to achieve the desired values. As such, the presented setup demonstrates a scalable and robust solution for the tailored synthesis of supported nanoparticles.

### Material Characterization

For TEM characterization, Cu nanoparticles were collected directly from the aerosol upstream of the bubble column by inserting carbon-coated TEM grids into the particle flow. This method allows the determination of the particle size generated by the spark ablation process. The aerosol subsequently enters the bubble column, where the particles are deposited onto Vulcan carbon (VC). A control experiment was performed to confirm that the size distribution of the particles collected in this way aligns with the particle size distribution after the bubble column/post-treatment procedure (see the Supporting Information). For the commercial nanoparticles, the Cu/VC were dispersed in ethanol, drop-cast onto the glassy carbon electrode, and their size was confirmed by SEM. X-ray photoelectron spectroscopy (XPS) measurements were performed on the drop-cast samples using a Kratos Axis Supra instrument, with binding energies calibrated to the C 1s peak (see the Supporting Information).

### Electrode Preparation

To make electrode samples, a nanoparticle ink was prepared and drop-cast onto a glassy carbon electrode (GCE). To mix the nanoparticle ink, 1.5 mL of isopropanol and 3 μL of Nafion solution were added to the nanoparticle (Cu/VC) suspension after evaporation or to the commercial nanoparticles (Cu/VC). Three commercial nanoparticle (Cu/VC) batches were purchased from Premetek: T40A010 (*d* = 15 nm), T40A100 (*d* = 25 nm), and T40A400 (*d* = 40 nm). Prior to deposition, the GCE was cleaned with ethanol, polished with alumina paste (1, 0.3, and 0.05 μm), thoroughly rinsed with Milli-Q water, and finally air-dried. Subsequently, 10-80 μL, depending on the desired mass loading, of the freshly prepared ink was drop-cast onto the surface of the GCE using a micropipette. The mass loading was controlled by adjusting the drop-cast volume. Afterward, the ink was dried under vacuum at 70 °C for approximately 15 min (Figure 1e).

### Electrochemical Measurements

For size-dependent oxidation studies in the following sections, an SSC reference electrode was employed and calibrated before each measurement session (see the Supporting Information). The deviation in these calibrations over 170 h was ±1 mV. The calibration value was used to calculate the potential vs. RHE from the SSC electrode measurement.

Before each experiment, the working electrode was immersed in the electrolyte under potential control. The cyclic voltammograms (CVs) were acquired from 0.2 to 0.8 V (vs. RHE) in 0.1 M HClO_4_ at a scan rate of 1.0 mV/s. Conductive filter substrates coated with deposited nanoparticles or glassy carbon electrodes coated with Cu NPs of different sizes were tested. Figure 2c shows a typical CV. The oxidation of Cu in acidic aqueous environments may proceed through Cu^+^/Cu_2_O intermediates before forming Cu^2+^. The anodic peak used for sizing is assigned to the overall oxidation of the Cu nanoparticles, likely involving Cu^+^/Cu_2_O intermediates rather than an exclusively direct reaction: Cu^0^ → Cu^2+^ + 2e^–^. The double-layer charging current was evaluated from the second anodic sweep, where no faradaic oxidation occurs. The capacitive current was found to be at least three orders of magnitude smaller than the faradaic oxidation peak observed in the first sweep. Consequently, the integrated charge is dominated by the faradaic oxidation of Cu nanoparticles, and the contribution from double-layer charging is negligible (<0.1%) for the purpose of mass loading normalization (Supporting Information, Figure S15). As in the work by Ivanova and Zamborini, a slow scan rate of 1 mV/s was selected to ensure electrochemical reversibility and planar diffusion conditions (overlapping diffusion profiles).^1^ At an applied scan rate of 1 mV/s, the diffusion layer thickness (*δ*) should be ∼370 μm over the ∼35 s time that it takes to oxidize the Cu NPs (peak width: 35-70 mV). The diffusion layer thickness (*δ*) is significantly greater than the average interparticle spacing, calculated to be in the order of ∼10^2^ nm; thereby, ensuring a planar diffusion regime (see the Supporting Information). Under the employed conditions (Ar-saturated 0.1 M HClO_4_, 1 mV/s), the typical peak current was approximately 20 μA, and the uncompensated resistance was about 8 Ω, corresponding to an estimated *iR* drop of only around 0.16 mV. This value is negligible relative to the observed size-dependent shifts in oxidation peak potential.

**Figure 2:**
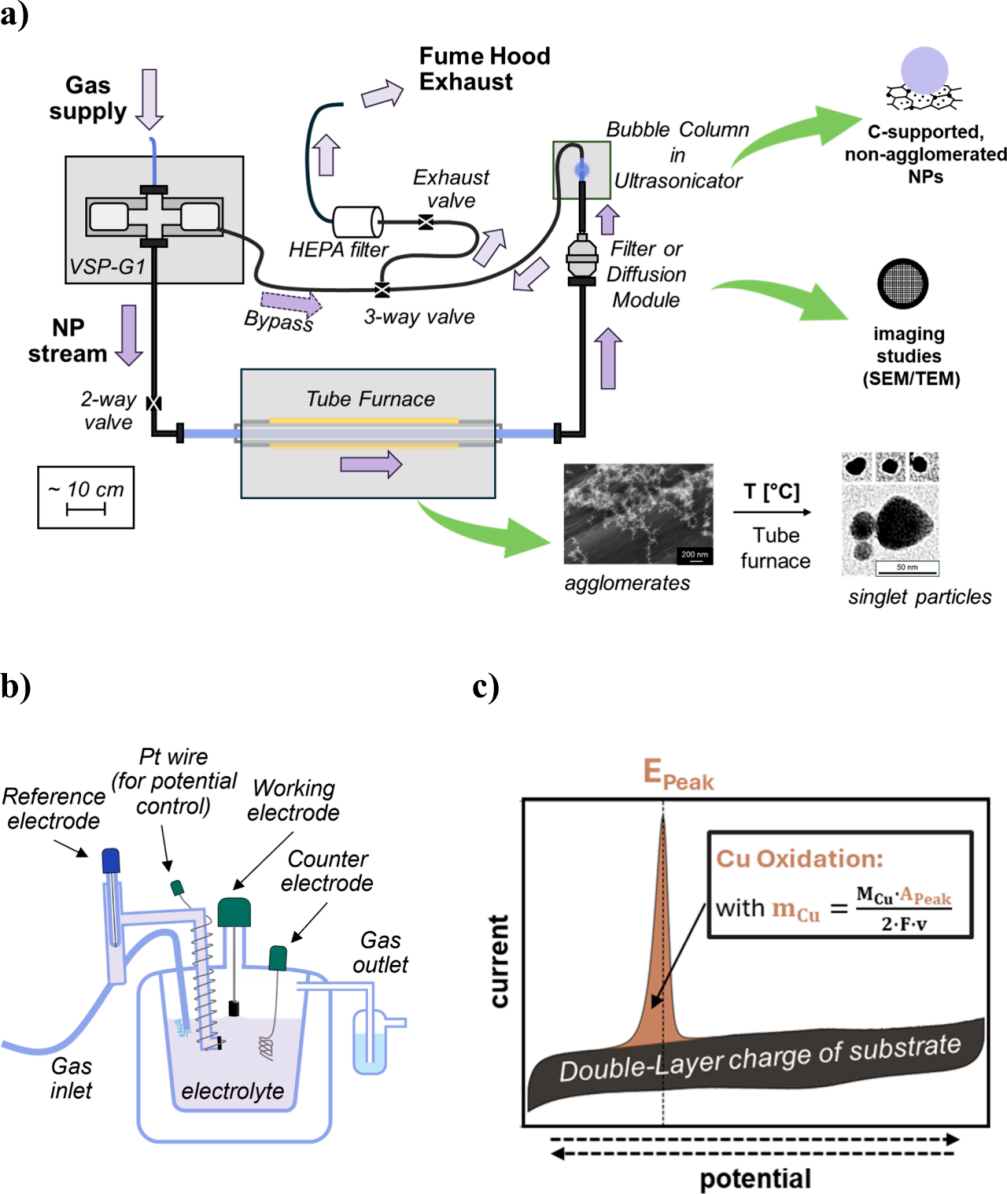
Schematic description of the synthesis and measurement setup used in this study. (a) Developed and employed spark ablation setup, including VSParticle Spark Ablation Generator (VSP-G1), tube furnace, and bubble column. (b) Electrochemical cell with working, reference, and counter electrodes (WE, RE, and CE), as well as a Pt wire coiled around the Luggin capillary used for keeping the WE under potential control when immersing into the electrolyte. (c) Exemplary CV of the oxidation of Cu NPs recorded in Ar-saturated 0.1 M HClO_4_ with a scan rate of *v* = 1 mV/s. The mass loading of Cu NPs on the electrode can be calculated by using the area under the oxidation peak *A*_peak_ and applying Faraday’s law (*F*: Faraday constant, *M*_Cu_: Molar mass of Cu).

## Results and Discussion

### Nanoparticle Synthesis and Characterization

The spark ablation strategy was elaborated to produce Cu nanoparticles suitable for size-dependent oxidation studies, aiming to yield nanoparticles with approximately spherical morphologies and minimal agglomeration across a broad size range (see the Supporting Information). Prior to electrochemical measurements, the particles were characterized using either SEM or TEM. To enable the extraction of nanoparticles from the gas stream for subsequent electrocatalytic experiments, we initially attempted to immobilize the nanoparticles using the VSP-G1 filtration module and a non-conductive filter. Due to the inherently low nanoparticle yield when using Cu as the electrode material, this approach proved unsuitable for our application. In practice, this approach either resulted in insufficient quantities of deposited nanoparticles to recover from the filter or yielded Cu NPs with a broad size distribution and excessive agglomeration (Supporting Information, Figures S4 and S5).

As an alternative, we implemented a direct filtration method using a conductive, porous substrate, such as carbon paper or carbon cloth, as a filter material.^35^ (Supporting Information, Figures S6 and S7). While this approach theoretically enables direct deposition onto a conductive substrate that can later be used as a working electrode, it proved suboptimal for our electrocatalytic applications. Specifically, a lack of uniformity in the deposited mass loading across the filter surface was observed. In addition, it frequently resulted in significant nanoparticle agglomerations on the filter, affecting the measured oxidation peaks and imposing a limit on the maximum achievable mass loading. Consequently, the bubble column approach was adopted. As shown in Figure 3a, this final bubble column approach, combined with a gas flow rate of 8 L/min, yields well-defined primary particles with diameters of approximately 4 nm.

**Figure 3:**
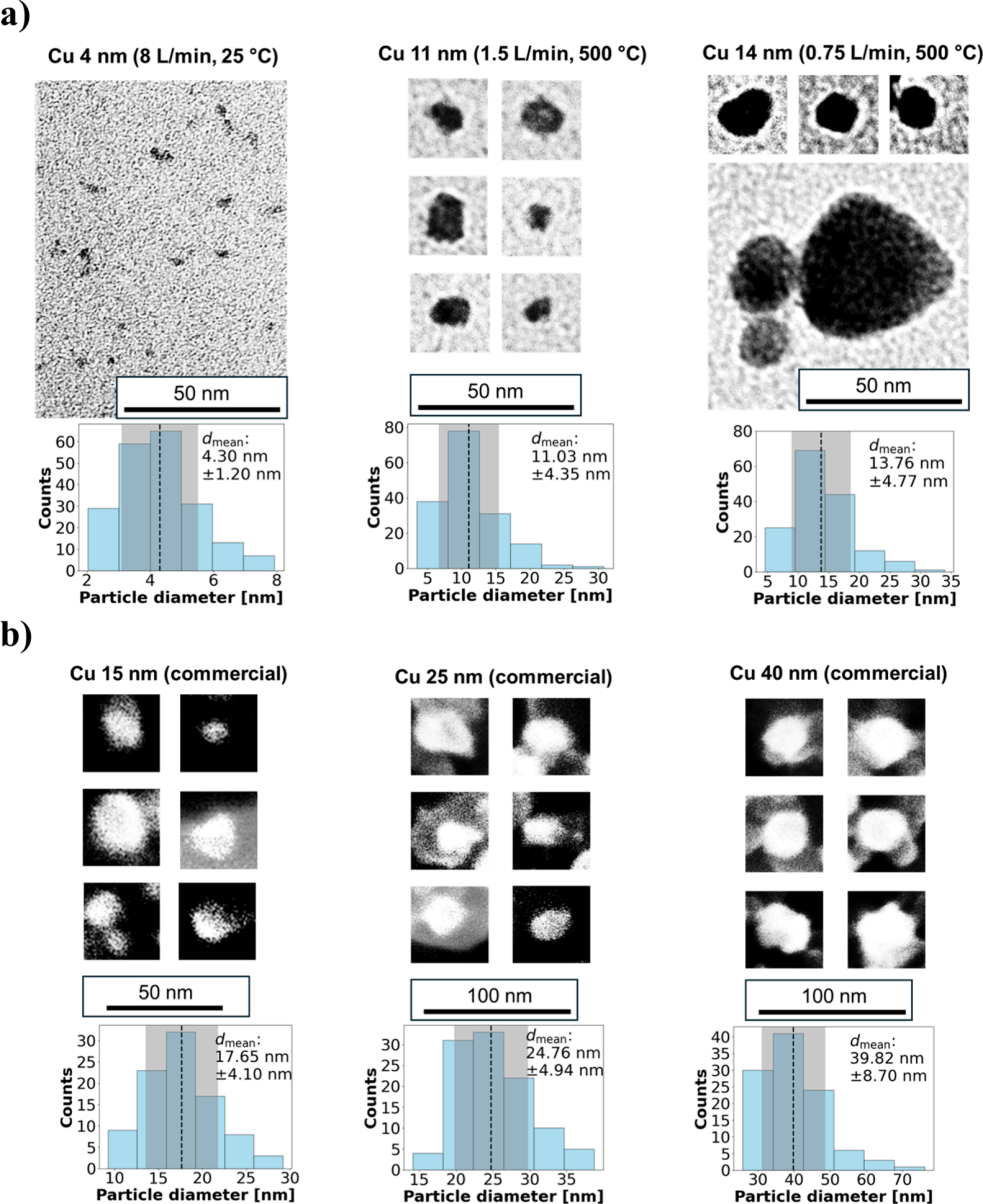
Characterization of Cu NPs with SEM and TEM. (a) Example TEM pictures of Cu NPs produced via the spark ablation setup and corresponding particle size distributions of the TEM analysis. (b) Example SEM pictures of commercial Cu NPs and the respective particle size distributions of the SEM analysis.

As reported in previous studies, gas flow rates exceeding approximately 5 L/min predominantly yield so-called primary particles.^29^ However, for the purpose of our study, we also required nanoparticles with diameters exceeding 5-6 nm, demanding the use of flow rates below 5 L/min. Operating at such low flow rates led to the formation of agglomerates with poorly defined size and morphology (Supporting Information, Figure S7). A possible solution to avoid this problem is in-flight annealing.

This approach has been successfully demonstrated for metals such as Au, Pt, and others.^32,36–38^ We also verified the effectiveness of in-flight annealing for Cu nanoparticles. As shown in Figure 3a, in-flight annealing enables the production of nanoparticles with diameters above 5 nm and more uniform, spherical morphologies, particularly under low-flow conditions that otherwise promote uncontrolled agglomeration. For lower gas flow rates and, therefore, larger nanoparticles, diffusion toward the TEM grid is reduced. The fact that the output of the VSParticle device is approximately constant and independent of the particle size further decreases the particle densities on the TEM grids (see the Supporting Information). Increasing the deposition time provided only limited compensation due to agglomeration effects (Supporting Information, Figure S11).

While both in-flight annealing and bubble column-based nanoparticle collection have been individually reported in the literature,^33,38^ to the best of our knowledge, this study presents the first integrated combination of these components into a unified and novel spark ablation/bubble column setup, specifically designed for producing carbon-supported nanoparticles for electrocatalytic applications.

Using this final configuration, we successfully produced Cu NPs with well-defined, sphere-like shapes and three distinct size distributions. The bubble column facilitated the deposition of supported Cu NPs without any signs of agglomeration, irrespective of the deposition duration. Table 1 summarizes the key process parameters and the resulting Cu NP radii. Corresponding TEM micrographs and particle size distributions are shown in Figure 3a. To assess the surface oxidation state of the as-prepared electrodes with the Cu/VC nanoparticles, XPS measurements were performed (Supporting Information, Figure S14). Analysis of the Cu LMM Auger region reveals a Cu^0^/Cu^+^ surface composition (Cu^0^:Cu^+^ ≈ 45.3 : 54.7), while no features attributable to Cu^2+^ are observed. These results indicate the absence of Cu^2+^ species and are consistent with mild surface oxidation upon air exposure, confirming that the particles are not significantly oxidized during synthesis. A quantitative analysis of interparticle distances shows that the average nearest-neighbor distance significantly exceeds the particle diameter, with less than 4% of particles in direct contact, confirming the largely non-agglomerated nature of the nanoparticle ensemble.

**Table 1:** Overview of Investigated Cu NP Samples (Synthesized via the Spark Ablation Setup and Commercial Samples)^a^

sample	diameter [nm] SEM/TEM	peak potential [mV] vs. RHE	full width at half-max [mV]	charge (*Q*) under the peak [mC]
8.0 L/min (25 °C)	4.3 (± 1.2)	316 (± 1)	41.9 (± 0.3)	1.19 (± 0.04)
1.5 L/min (500 °C)	11.0 (± 4.4)	326 (± 2)	34.3 (± 0.5)	1.16 (± 0.26)
0.75 L/min (500 °C)	13.8 (± 4.8)	329 (± 1)	40.0 (± 5.2)	0.82 (± 0.12)
commercial 15 nm	17.7 (± 4.1)	337 (± 3)	58.3 (± 3.6)	1.54 (± 0.03)
commercial 25 nm	24.8 (± 4.9)	351 (± 1)	49.1 (± 0.2)	1.50 (± 0.21)
commercial 40 nm	39.8 (± 8.7)	367 (± 1)	57.4 (± 9.7)	1.51 (± 0.04)

a The table shows the mean values of all measurements per sample.

Since 0.75 L/min represents the lowest achievable gas flow rate, defined singlet particles with diameters of approximately 15 nm constitute the largest size attainable with the spark ablation setup for Cu. Therefore, particles with larger diameters were purchased from Premetek and characterized using SEM (Figure 3b). The mean particle diameters (see Table 1) are consistent with the supplier’s values.

### Size-Dependent Oxidation of Cu Catalyst Nanoparticles

The primary motivation was to produce non-agglomerated Cu NPs immobilized on VC with controlled morphologies and size distributions. These non-agglomerated Cu NPs of different sizes were required to investigate the size-dependent oxidation behavior of Cu NPs. Plieth proposed a 1/*r* dependency of the oxidation potential of NPs.^17^ However, Kolb and Simeone have shown that tiny clusters of Cu on Au exhibit enhanced stability against electrochemical oxidation compared to the bulk metal, in contrast to Plieth’s predictions.^17,20^ Hence, it is essential to investigate the oxidation behavior of Cu NPs of different sizes using electrochemical methods to gain deeper insight into the influence of the particle size on their oxidation potential. For this purpose, this study adapts the experimental methodology previously employed by Ivanova and Zamborini for silver nanoparticles.^1^ CV measurements were conducted using GCEs with supported Cu NPs of different sizes. The electrochemical characterization was performed in 0.1 M HClO_4_ electrolyte in the potential range of 0.2 to 0.8 V (vs. RHE) at a scan rate of 1.0 mV/s. The anodic current is attributed to the oxidation of the Cu nanoparticles, which may proceed via intermediate Cu^+^/Cu_2_O species, while the overall oxidation corresponds to a two-electron process leading to Cu^2+^ under the employed conditions. The respective distinct electrochemical oxidation peaks of the Cu NP samples produced with the Spark Ablation Setup, as well as the oxidation peaks of the commercial Cu NP samples, are depicted in Figure 4.

**Figure 4:**
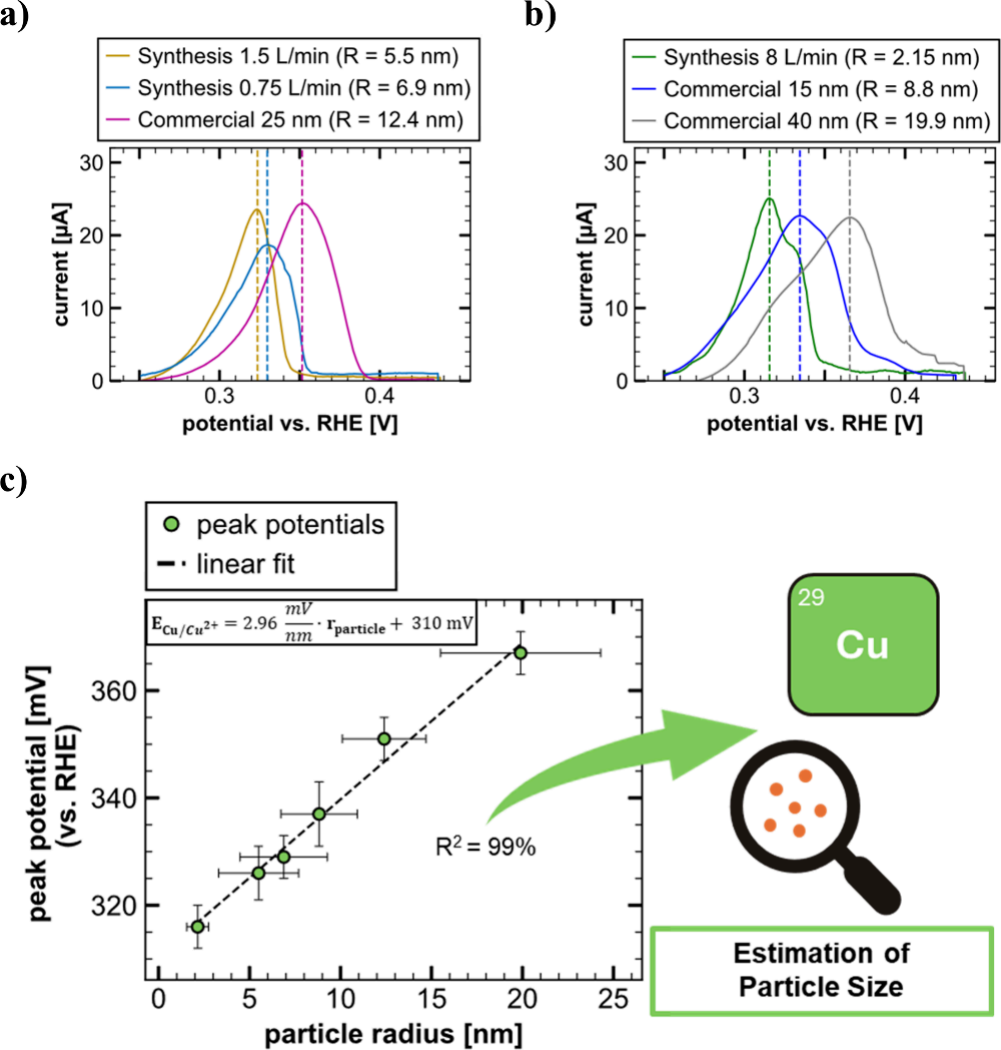
Size-dependent oxidation of Cu NPs. (a, b) CVs obtained in 0.1 M HClO_4_ electrolyte at 1.0 mV/s of GC electrodes coated with Cu NPs of different sizes/radii. (c) Linear correlation (*R* ^2^ = 0.99) between particle radius and peak potential. The average *E*_p_ values shift positively with increasing Cu NP size (radius). The established linear correlation can be used to estimate the particle size of Cu NPs by direct electrochemical measurements.

Following the methodology suggested by Ivanova and Zamborini,^1^ a slow scan rate of 1 mV/s was employed to ensure electrochemical reversibility and establish planar diffusion conditions with overlapping diffusion profiles. Consistent with the observations of Ivanova and Zamborini,^1^ our experimental results demonstrate that for one size of Cu NPs, the potential at which the oxidation peak can be observed (*E*_p_) exhibits a direct proportional relationship with the logarithm of the mass loading and therefore the area under the peak ln (*Q*) (Supporting Information, Figure S16). This aligns with the expected behavior for reversible oxidation kinetics.

To eliminate mass loading effects, the total mass loading was kept constant, so that the charge under the peak *Q* was approximately 1.3 mC (averaged across all measurements). As shown in Table 1, *Q* ranges from ∼0.82 mC at the lowest to ∼1.54 mC at the highest mass loading. This corresponds to a maximum Δln (*Q*)_max_ of approximately 0.6 and a variation around the mean value of about 0.3. Using the direct proportional relationship between ln (*Q*) and *E*_p_ (Supporting Information, Figure S16), the error resulting from variations in mass loading can thus be estimated to be 0.3 × 10.4 mV ≈ 3 mV. In addition, the potential influence of the carbon support was also evaluated. Varying the Cu loading on Vulcan carbon (wt % Cu on VC) while maintaining a constant electroactive Cu mass loading on the electrode did not result in a systematic shift of the oxidation peak potential (Supporting Information, Figure S17), indicating that support-induced effects are not dominant under the present conditions.

Along with the average diameters of the various Cu NPs obtained from SEM/TEM analyses, Table 1 presents the corresponding average *E*_p_ values, full width at half maximum (FWHM), and peak charge determined from the CV measurements (based on at least two reproducible measurements per size). The error values indicate variations in peak potential across the CV measurements for each sample. The average peak potential values shift positively with increasing Cu NP radius. Overall, *E*_p_ shifts ∼51 mV from the smallest sample (*r* = ∼2.2 nm) to the largest sample (*r* = ∼19.9 nm), indicating that there is an effect of size-dependent oxidation for all investigated Cu NPs. In addition, the oxidation peak width (FWHM) shows an approximately linear increase with nanoparticle size dispersity (*δ*_radius_), indicating that broader size distributions lead to broader oxidation peaks (Supporting Information, Figure S18).

Under idealized conditions (reversible system, constant coverage, planar diffusion), the previous theory by Compton, based solely on diffusion, predicts that the oxidation peak potential, *E*_p_, should be independent of particle size.^18^ However, real nanoparticle systems may deviate from these ideal conditions, and size-dependent shifts in *E*_p_ have been reported that do not necessarily reflect changes in the intrinsic thermodynamic standard potential (*E*°).^19^ Therefore, the observed negative shift in *E*_p_ with decreasing particle size is consistent with previously reported size-dependent electrochemical behavior of metal nanoparticles.^1^ While such shifts may be qualitatively consistent with a size-dependent change in the standard redox potential, as predicted by Plieth and Henglein and discussed by Redmond,^17,39,40^ the present data do not allow an unambiguous assignment of the peak shift solely to a change in the standard redox potential (*E*°). Additionally, it should be noted that the peak potential measured in cyclic voltammetry is not identical to the thermodynamic redox potential and may also reflect kinetic and interfacial contributions. While size-dependent electrochemical behavior of nanoparticles has been reported for systems such as Au,^22–26^ Ag,^1^ and Pd^27^ in previous voltammetric studies, and to a more limited extent for Cu nanoparticles,^28^ systematic investigations of supported Cu nanoparticle ensembles and their potential use for electrochemical size analysis remain comparatively scarce.

Figure 4c presents peak potential as a function of Cu nanoparticle radius (determined from SEM/TEM measurements). The total error in peak potential for each sample is the sum of the errors from the mass loading effect, as previously described, and the variation in peak potential (see Table 1).

Contrary to Plieth’s theoretical predictions,^17^ our results demonstrate a strong linear relationship (*R* ^2^ = 0.99) rather than the expected 1/*r* dependence. The linear relationship can be described by ECu,ox=2.96mVnm×rparticle+310⁢ mV . The linear regression, parameter uncertainties, standard errors (SE), and 95% confidence intervals (CI) are calculated from the weighted least-squares regression (Supporting Information, Table S3). The calibration curve includes nanoparticles produced by spark ablation as well as commercially available materials synthesized by different routes. The fact that these particles follow the same *E*_p_ -radius trend indicates that the observed dependence is primarily determined by particle size rather than the synthesis method. We also tried to apply the model of Plieth^17^ to our data by using (a) only the oxidation potential of the bulk material *E*_bulk_ as a fitting parameter and using the surface tension *γ* of Cu in vacuum (2190 erg/cm^2^), and (b) by treating *E*_bulk_ and the surface tension *γ* of Cu as fitting parameters. The second approach (b) would be reasonable, as *γ* in this context cannot be regarded as the bare surface tension but rather as an effective ″electrochemical surface stress″ renormalized by the electrolyte, double layer structure, and oxide formation processes. However, neither fit was in good agreement with the experimental data (Supporting Information, Figure S19).

Ivanova and Zamborini were also unable to reconcile their experimental data on the oxidation of Ag nanoparticles with the thermodynamic model predicted by Plieth;^17^ however, they did not propose an alternative correlation.^1^ Therefore, we re-evaluated their data and found that in Ag nanoparticle systems in the same particle size range as our Cu NPs (*d* = 4–40 nm), the oxidation potential also strongly correlates linearly with the Ag nanoparticle radius (*R* ^2^ = 0.96). (Supporting Information, S20) The correlation can be described by *E*_Ag,ox_ = 7.98 mV/nm × *r*_particle_ + 235 mV. Classical thermodynamic treatments predict a 1/*r* dependence of nanoparticle redox potentials for idealized, monodisperse systems under equilibrium conditions. However, the anodic peak potential measured in cyclic voltammetry does not directly correspond to the thermodynamic redox potential (*E*°), but instead reflects a dynamic, kinetically influenced ensemble oxidation process rather than the equilibrium potential of individual particles. As a result, deviations from the ideal 1/*r* scaling can arise, and over a limited size range, an approximately linear dependence on particle radius may emerge as an effective description of the ensemble response. The observation of an approximately linear correlation between oxidation peak potential and particle radius for both Cu and Ag under comparable conditions suggests that this behavior reflects the ensemble electrochemical response of nanoparticle populations rather than the strict thermodynamic scaling of individual particles.

It should be noted that the anodic peak potential measured during cyclic voltammetry can, in principle, be influenced not only by size effects but also by kinetic factors, mass transport, and interfacial phenomena. However, all nanoparticle samples were measured under identical electrochemical conditions (electrolyte composition, scan rate, and electrode configuration), and the Cu mass loading was normalized between samples. Under these controlled conditions, such effects are expected to influence all measurements in a comparable manner. The systematic shift in oxidation peak potential with nanoparticle size, therefore, reflects a genuine size-dependent trend rather than variations in the electrochemical measurement conditions. Because the nanoparticle ensembles exhibit finite size dispersion, the observed oxidation peaks reflect the collective electrochemical response of particles with different sizes rather than a single well-defined particle size. Accordingly, the peak potential *E*_p_ should be interpreted as an effective ensemble descriptor. The well-defined and approximately symmetric peak shapes, together with the strong linear correlation with the independently determined mean particle radius, indicate that the electrochemical response is not dominated by a small fraction of particles but instead reflects the overall nanoparticle population.

The *E*_p_-radius relationship reported here was established under a defined set of electrochemical conditions (Ar-saturated 0.1 M HClO_4_ electrolyte, 1 mV/s scan rate, and a fixed catalyst ink formulation). Variations in electrolyte composition, scan rate, or catalyst layer composition may influence the absolute peak potential. Therefore, the present correlation should be regarded as a calibration valid within the employed experimental framework.

Ultimately, the established linear correlations for Cu and Ag can be used to estimate the particle size of Cu and Ag NPs between 4 and 40 nm in diameter via direct electrochemical measurements, without the need for high-cost techniques such as TEM.

## Conclusions

In this study, we developed a scalable approach for the synthesis of nanoparticles using a plasma-based physical method. The presented spark ablation strategy enables the direct production of high-purity, non-agglomerated copper (Cu) nanoparticles immobilized on a carbon support, without the need for surfactants or the often environmentally unfriendly reducing agents commonly used in conventional wet-chemical routes. The custom-built setup enables high collection efficiency and allows for continuous, scalable production with tunable mass loading. The integration of a tube furnace further expands the attainable particle size range, enabling the formation of spherical Cu nanoparticles up to 15 nm in diameter without agglomeration. The employed bubble column collector ensures homogeneous particle distribution on the support material, enabling continuous, scalable nanoparticle production without agglomerations, overcoming the limitations of traditional filter-based systems.

To address the challenge of nanoparticle size characterization, we introduced a straightforward electrochemical method based on anodic potential sweeps to determine particle size via size-dependent oxidation potentials. For the synthesized Cu nanoparticles, a strong linear relationship (*R* ^2^ = 0.99) was observed between the particle radius and the oxidation peak potential. This correlation was further validated using literature data on Ag nanoparticles (*R* ^2^ = 0.96), confirming the approach’s probable broader applicability. Attempts to model the experimental data and literature data using existing theoretical frameworks for size-dependent oxidation were unsuccessful, suggesting that current models do not fully capture the observed electrochemical behavior.

Overall, our findings provide new insights into the size-dependent oxidation of metallic nanoparticles and demonstrate that the particle size of approximately spherical Cu nanoparticles can be reliably estimated using simple, low-cost electrochemical measurements. This electrochemical approach offers an attractive alternative to resource-intensive microscopy techniques such as Transmission Electron Microscopy.

## Supplementary Material



## Notes

Additional experimental details, materials and methods, electrochemical measurements, data analysis and fitting procedures, and supplementary figures and tables (PDF)
