# Supplementary material for: Size-Dependent Oxidation of Copper Nanostructured Electrocatalysts Produced by Spark Ablation
Source: ACS Electrochem. 2026 May 18;2(6):1397–405. doi: 10.1021/acselectrochem.6c00062 (PMC13244637; doi:10.1021/acselectrochem.6c00062)
Supplement: Supplementary file 1 [file ec6c00062_si_001.pdf]

Size-dependent Oxidation of Copper Nano-structured Electrocatalysts Produced by Spark Ablation

Supporting Information

*Johannes Sterzinger,[a] Bogdan Gulie,[a] Vincenz Maier,[a] Tim Steeger,[a] Carsten Peters,[b] Nikolaos Patelis,[a] Elena Gubanova,[a] Marc Willinger,[b] Aliaksandr S. Bandarenka [a,c,] **

[a] Physics of Energy Conversion and Storage, Technical University of Munich, Physics Department, James-Franck-Str. 1, 85748 Garching, Germany

[b] Chair of Electron Microscopy, Technical University of Munich, Chemistry Department, Lichtenbergstraße 4, 85748 Garching, Germany

[c] Catalysis Research Center TUM, Ernst-Otto-Fischer-Str. 1, 85748 Garching, Germany

*Corresponding author e-mail: bandarenka@ph.tum.de

Contents

Overview of NP Immobilization Methods (without bubble column approach)	S3
Comparison of Diffusion, Filtration, and Impaction Methods	S4
Indirect Deposition (Filtration with non-conductive substrate)	S5
Direct Deposition: Non-uniform Particle Distribution with Filter Module	S7
Direct Deposition: Comparison of different substrates	S9
Influence of annealing on Cu NP size and shape	S13
Bubble Column Design	S14
Estimation of the Cu NP mass loading on VC via TGA	S15
TEM Analysis with diffusion	S16
TEM Analysis after Bubble Column Collection/Post-Treatment	S17
XPS Characterization	S19
Reference Electrode Calibration	S21
Oxidation Measurements of Cu NPs	S22
Assumption of Planar Diffusion	S24
Effect of Mass Loading on the Oxidation Peak Potential	S25
Effect of Loading (w.-% Cu/VC) on the Oxidation Peak Potential	S27
Effect of Nanoparticle Size Dispersity on Oxidation Peak Width	S28
Model by Plieth (Fitting)	S29
Linear Fit for Copper and Silver Nanoparticles (data from Ivanova et al.)	S30
References	S32

Optimization of Nanoparticle Synthesis via Spark Ablation

Overview of NP Immobilization Methods (without bubble column approach)

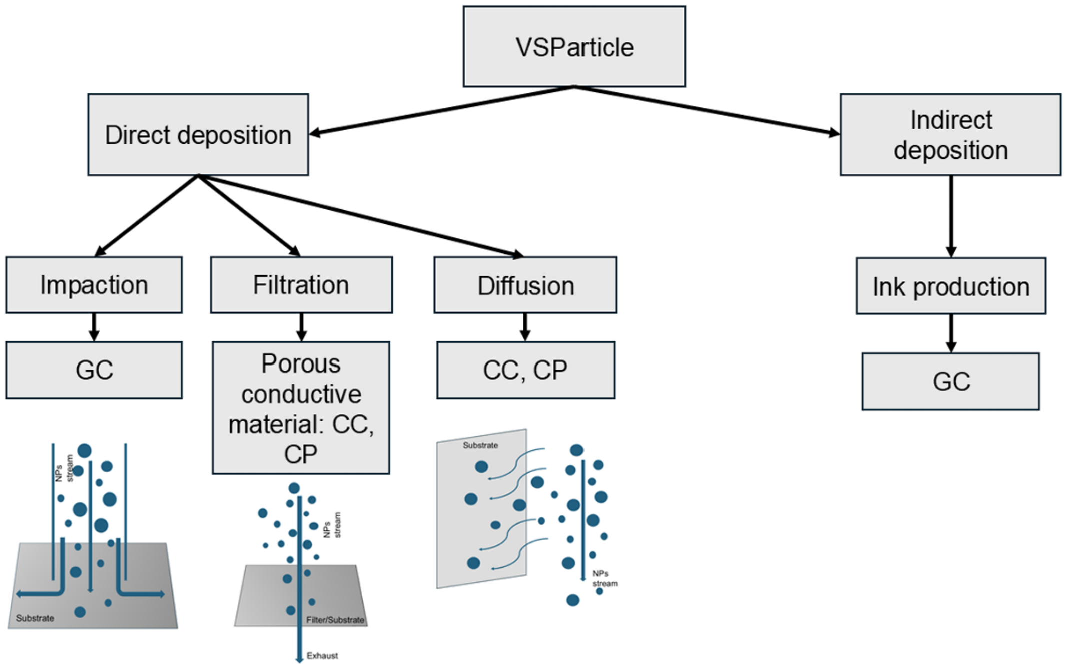

Figure S1: A diagram of the tested deposition methods and substrates. Direct deposition requires conductive substrates, such as carbon cloth (CC), carbon paper (CP). For the indirect deposition, a non-conductive filter substrate can be used. An ink can be made from these collected particles and drop-cast onto a glassy carbon (GC) electrode.

Comparison of Diffusion, Filtration, and Impaction Methods

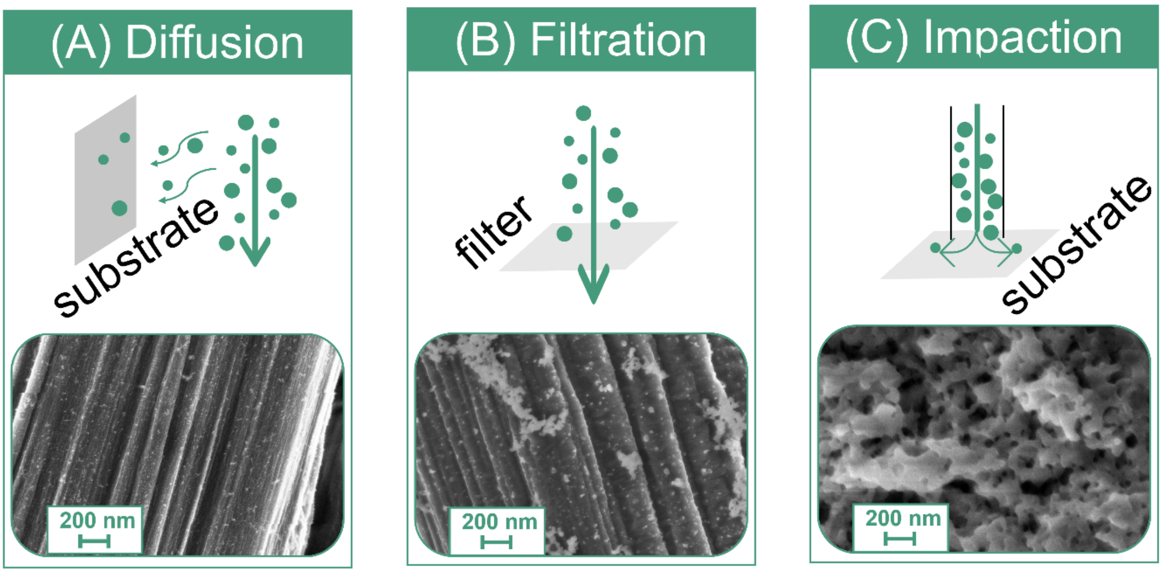

Figure S2: Comparison of SEM images of samples produced with the different methods using the VSPARTICLE modules. (diffusion: 3 L/min, 1 kV, 10 mA, 2.5 h; filtration: 3 L/min, 1 kV, 9 mA, 20 min, CC; impaction: 0.1 L/min, 1.3 kV, 10 mA, 60 min, 0.1 mm nozzle).

While the diffusion module enables the collection of well-separated, individual particles, it has inherent limitations. Its collection efficiency is low, it is not suitable for upscaling, and particle adhesion to the substrate is poor, particularly in the context of electrochemical measurements. Nevertheless, it is advantageous for preparing nanoparticles on TEM grids for subsequent analysis. The advantages and limitations of the filter module will be discussed below. Finally, the impaction module produces nano-sintered structures.

Indirect Deposition (Filtration with non-conductive substrate)

To enable the collection of nanoparticles from the gas stream for subsequent electrocatalytic measurements, we initially employed the filtration module of the VSP-G1 for nanoparticle immobilization. However, owing to the intrinsically low nanoparticle yield obtained when using copper as the electrode material, this approach proved unsuitable for the present study. In the indirect deposition method, which was applied in this case, nanoparticles were first collected on a non-conductive filter, then processed into an ink, and finally drop-cast onto a conductive substrate intended for use as an electrode. This approach either led to an insufficient quantity of deposited nanoparticles to recover from the filter or yielded Cu NPs exhibiting a broad size distribution and excessive agglomeration (see Figures S3, S4, S5).

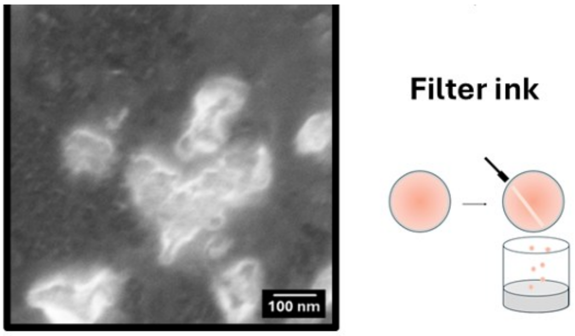

Figure S3: Indirect filter approach with subsequent ink production and drop casting onto GCE electrode. Spark Ablation Parameters: 0.75 L/min, 1.2 kV, 10 mA, 6 h, reaction chamber to filter module distance: 14 cm, 0.45 µm filter.

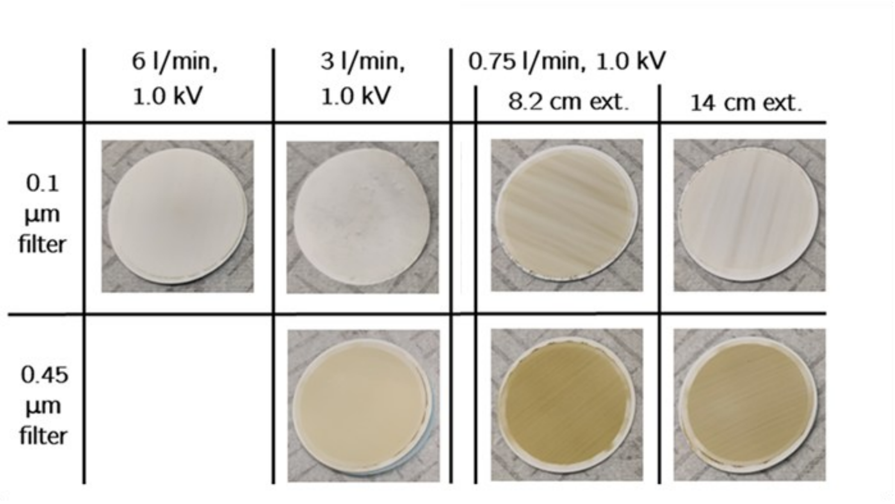

Figure S4: Indirect deposition was performed using filter materials of different pore sizes. The deposition efficiency can be evaluated visually by monitoring the color change of the filter material.

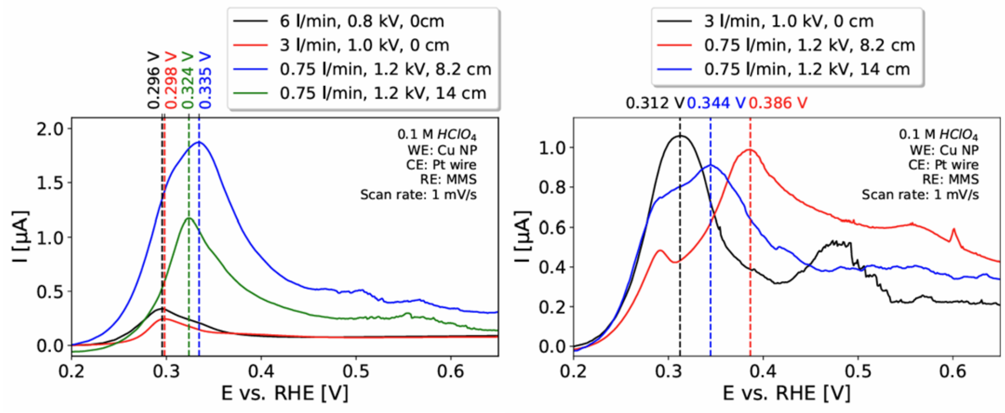

(a) 0.1 μm filter

(b) 0.45 μm filter

Figure S5: Electrochemical oxidation peaks of Cu NPs produced via indirect deposition. The results reveal significant particle agglomeration resulting from the collection process.

Direct Deposition: Non-uniform Particle Distribution with Filter Module

To replace indirect deposition, we implemented a direct filtration strategy in which conductive, porous substrates, including carbon paper or carbon cloth, served as the filter material.[1] To prepare electrode samples, Cu NPs were deposited onto conductive filter substrates (diameter of 46 mm), including carbon cloth, carbon paper, and graphite paper, and then punched into 14 mm discs (see Figure S6). However, this method has notable limitations, which are discussed in more detail in the subsequent sections. To ensure compatibility, all electrochemical measurements related to the spark ablation setup described in subsequent sections were conducted on the same day using either an SSC or MMS reference electrode. The separation between the reaction chamber and the filtration module was adjusted with different tube extensions (e.g., 8.2 cm, 14 cm) with the aim of enhancing aerosol mixing prior to filtration. The goal of this test was to improve the uniformity of particle deposition across the filter (e.g., minimizing edge-to-center gradients).

However, an inhomogeneous distribution of deposited mass loading was observed over the filter surface. A clear radial gradient in mass deposition was evident: working electrodes punched out from the center of the filter substrate exhibited consistently higher mass loadings compared to those taken from the edges.

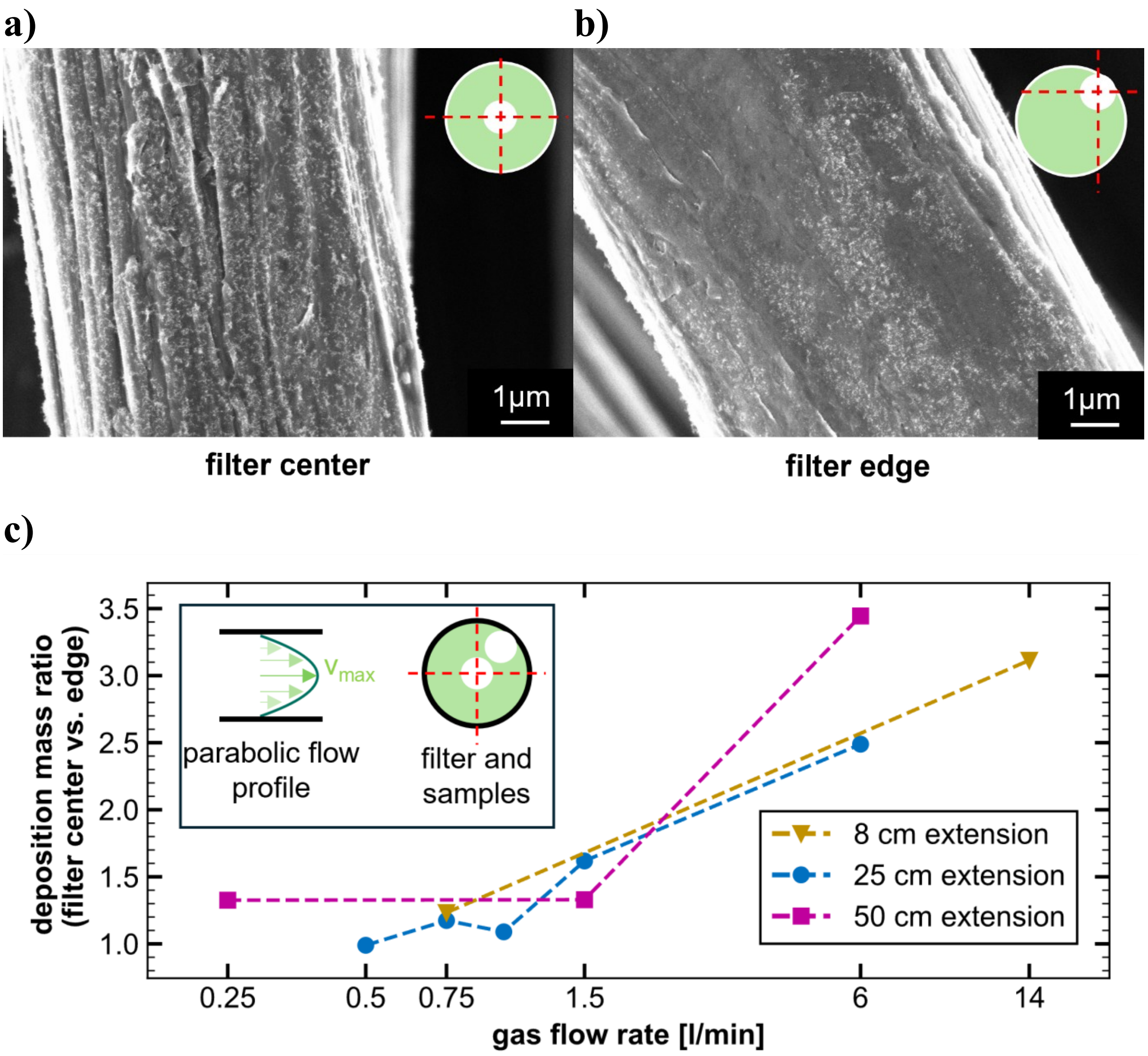

Figure S6: Substrates used in the filter module exhibit a non-uniform mass loading of particles on their surface. Sample electrodes (white) were punched out of the center as well as the edge regions of the carbon cloth filter (green) and tested. The higher the flow rate, the more non-uniform the mass loading on the filter. (a) SEM image of a carbon cloth sample taken from the middle of the carbon cloth filter substrate vs. (b) SEM image of a carbon cloth sample taken from the edge of the carbon cloth filter substrate. (c) Mass loading ratios of the samples from the middle and the edge regions of the carbon cloth substrate used in the filter module.

This inhomogeneity becomes increasingly pronounced at higher gas flow rates. As illustrated in Figure S6c, the center-to-edge mass loading ratio remains close to unity under low-flow conditions, but increases significantly with higher flow rates. This behavior is attributable to the transition in flow regime: at elevated flow rates, the system approaches laminar flow conditions, while turbulent flow predominates at lower flow rates. As described in the literature,

turbulent flow enhances mixing and promotes uniform particle distribution, whereas laminar flow results in minimal mixing, with nanoparticles predominantly following the streamlines of the flow.[2] Under laminar conditions, the characteristic parabolic velocity profile leads to preferential particle deposition at the center of the substrate (Figure S6a).

These electrochemical observations were further confirmed by scanning electron microscopy (SEM). At flow rates higher than 1.5 L/min, SEM images revealed a markedly higher nanoparticle concentration at the center of the filter compared to the edge (Figure S6b), consistent with the proposed deposition mechanism.

Direct Deposition: Comparison of different substrates

As described in the preceding section, initially, carbon cloth was employed as a conductive substrate for the direct deposition of nanoparticles via a filtration-based module. However, in addition to the previously discussed issue of non-homogeneous mass loading across the filter surface, we encountered difficulties in achieving controlled nanoparticle size distributions.

In theory, the nanoparticle size in spark ablation setups can be tuned by adjusting either the residence time of the particles in the post-reaction flow path or the energy of the initial plasma discharge, which is governed by the gap voltage.[3] Given the impracticality of modifying the tube length between experiments, the most accessible method for altering residence time is by varying the gas flow rate while keeping the tube length constant. As illustrated in Figure S7a, the nanoparticle size exhibits an inverse exponential dependence on gas flow rate, with higher flow rates yielding smaller particles. In contrast, the particle size decreases linearly with decreasing gap voltage.

Despite this expected relationship between gas flow rate and particle size, attempts to tune particle size by varying the gas flow rate (at constant voltage) using carbon cloth as the collection substrate failed to produce any observable changes in the oxidation peak position during potential scans (Figure S7b), which is expected to correlate with particle size, as discussed above. Considering that the substrate material might influence the outcome, the carbon cloth was substituted with carbon paper. This change of substrate caused distinct shifts in the oxidation peak position, indicating a successful variation in particle size.

To understand this discrepancy, it is essential to consider the structural differences between these two substrates. Carbon cloth is composed of woven bundles (tows) of carbon filaments, which form a fabric with relatively large interstitial voids between the tows, even discernible with the naked eye. In contrast, carbon paper consists of arbitrarily oriented carbon fibers bonded together by a polymeric binder, resulting in a more compact and homogeneous structure.

The large pores in the carbon cloth likely prevent accurate sampling of the nanoparticles transported in the gas stream, as the deposited particles do not represent the actual particle size distribution. The constant oxidation peak position observed across all flow rates at the same potential is lower than that using carbon paper. This observation suggests a preference for collecting smaller particles, regardless of the gas flow rate and the actual particle size distribution in the gas stream. The peaks presented in Figure S7c correspond to different mass loadings, as indicated by the varying area under each peak.

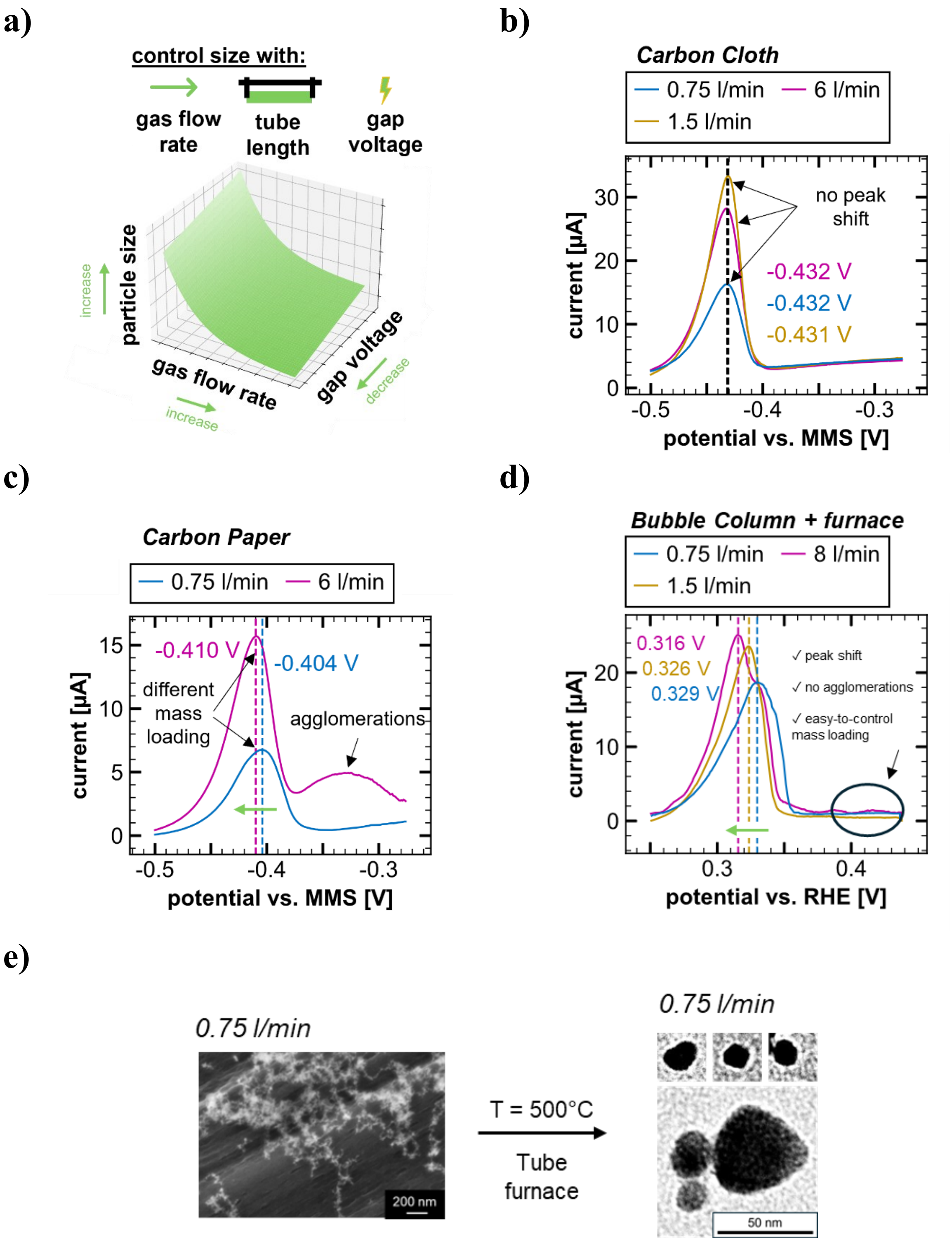

Figure S7: Comparison of conductive substrates in a filtration-based approach versus samples produced with the optimized bubble column approach. a) Effect of synthesis parameters on the nanoparticle size in spark ablation systems. Electrochemical oxidation peaks of Cu NPs synthesized at different gas flow rates using b) carbon cloth as filter substrate, c) carbon paper as filter substrate, and d) the bubble column approach + furnace for lower flow rates (0.75 L/min, 1.5 L/min). e) Effect of in-flight annealing on nanoparticle morphology.

As demonstrated later, the position of the oxidation peak is also influenced by the amount of mass loading. However, even when accounting for this effect, a more pronounced separation in peak potential would be expected; however, this was not the case.

In contrast, carbon paper enabled the deposition of nanoparticles with flow-rate-dependent size distributions, as reflected by distinct shifts in oxidation peak positions. These findings were further confirmed by experiments using graphite paper (see Figure S8), which, like carbon paper, has a dense structure without large voids and also demonstrated peak shifts in accordance with flow rate changes. Again, although mass loadings varied, taking into account the later described mass-loading-peak position correlation would lead to even more pronounced separation in the oxidation peak position.

Despite these improvements, the direct deposition approach ultimately proved to be unsuitable for our experiment. It frequently resulted in significant nanoparticle collection-induced agglomeration, affecting the measured oxidation peaks and the previously discussed central-edge deposition inhomogeneity. Besides, it imposes limitations on the maximum achievable mass loading, and is making it difficult to control mass loading overall.

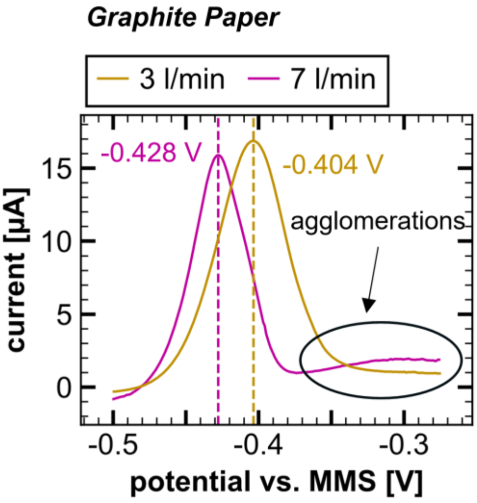

Figure S8: Electrochemical oxidation peaks of Cu NPs collected on graphite paper. As for carbon paper, a peak shift can be observed, but also the electrochemical response of collection-induced agglomerations.

Influence of annealing on Cu NP size and shape

According to the literature, agglomerates can be transformed into more uniform, spherical single particles through annealing.[4]

As shown in Figure S7e, a comparison between particles produced with and without the tube furnace demonstrates that in-flight annealing yields favorable results at a low flow rate, e.g., 0.75 L/min. Although many of the resulting particles exhibit an approximately spherical morphology under these conditions, clusters of such particles, partially fused, are also present. These clustered structures appear to decrease in number as the gas flow rate is increased up to 1.5 L/min, suggesting improved particle separation and reduced coalescence at higher flow rates.

In-flight annealing promotes the formation of nanoparticles exceeding 5 nm in diameter with more uniform and spherical morphologies, mitigating the uncontrolled agglomeration typically observed under low-flow conditions.

Bubble Column Design

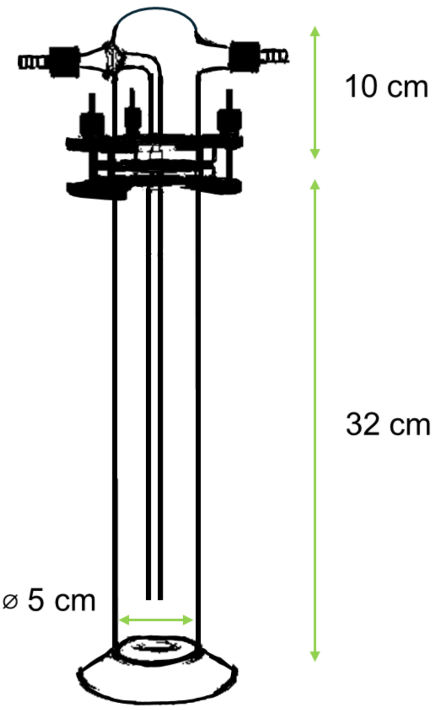

Figure S9: Dimensions of the employed bubble column.

The collection efficiency η of the bubble column can be predicted using the Pich and Schütz model[5]:

$$\eta_{BC} = 1 - \exp\left(-a \cdot h\right) \text{ with } a \approx 5.1 \left(\frac{D}{U_b \cdot d_b^3}\right)^{1/2},$$

where h represents the liquid column height, and a denotes the adsorption coefficient, which depends on the Brownian diffusion coefficient D, bubble rising velocity U_b, and bubble diameter d_b.

Based on this model, the critical design parameters for optimizing bubble column performance are the column dimensions and orifice diameter. The column dimensions determine both the water column height per unit liquid volume and the maximum achievable liquid column height,

while the orifice diameter strongly influences the resulting bubble diameter. To maximize collection efficiency, the design should prioritize maximizing the liquid column height (h) while minimizing the bubble diameter (d_b).

Estimation of the Cu NP mass loading on VC via TGA

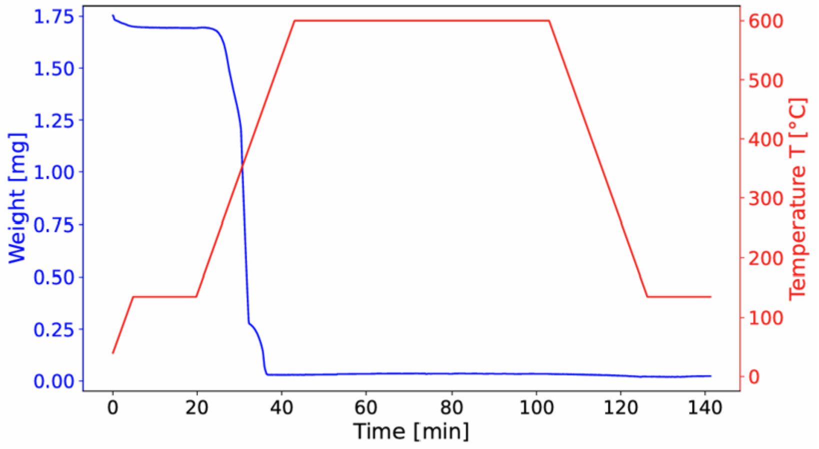

Figure S10: TGA measurement of the 0.75 L/min sample produced with the Spark Ablation setup.

TEM Analysis with diffusion

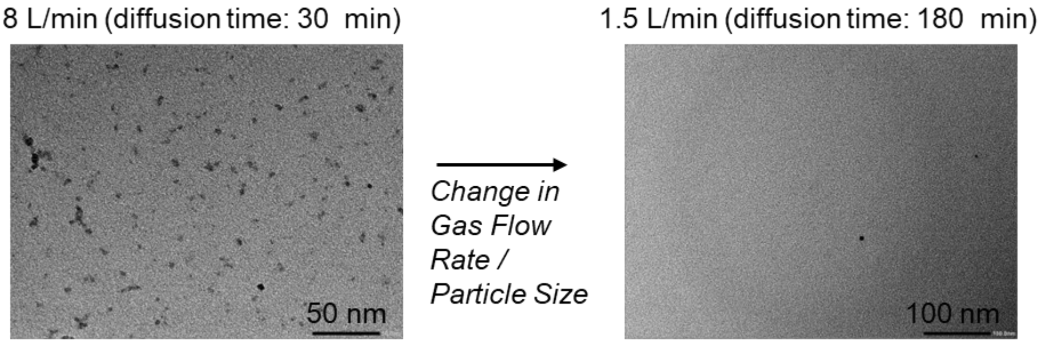

Figure S11: TEM images of Cu NPs produced at a gas flow rate of 8 L/min (25 °C) and 1.5 L/min (500 °C). The particle density on the TEM grids is strongly reduced with lower gas flow rates due to reduced diffusion towards the TEM grids.

With increasing nanoparticle diameter, diffusion towards the TEM grid within the diffusion module becomes less pronounced. In addition, the mass loading output of the VSParticle device is approximately constant, and the number of particles produced is governed by their size. As a result, larger particles yield a lower particle density on the TEM grids. Although the deposition time can be increased to compensate, this is only effective up to a certain extent, as prolonged deposition promotes agglomeration on the TEM grids. Consequently, despite extending the deposition time, the TEM images remained sparsely populated, necessitating the acquisition of a substantially larger number of micrographs.

TEM Analysis after Bubble Column Collection/Post-Treatment

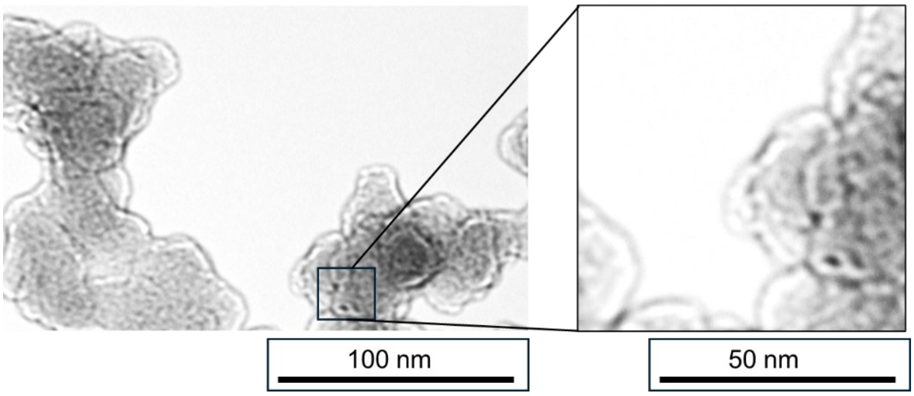

Figure S12: Example TEM pictures of Cu NPs supported on VC produced via the spark ablation setup/bubble column approach (8 L/min, 25°C). After the bubble column and post-treatment steps, the ink (without binder) was drop-cast onto TEM grids.

To verify whether the particle size is preserved during the bubble column and post-treatment steps, and to evaluate the nanoparticle dispersion on the support, a control experiment was performed using the smallest particle sample (8 L/min, 25 °C). After completion of the bubble column process and post-treatment, the catalyst ink (without binder) was drop-cast onto TEM grids and analyzed.

Representative TEM images of the Cu nanoparticles supported on Vulcan carbon are shown in Figure S12. The particles are well dispersed across the carbon support without significant agglomeration. To quantify the particle dispersion, an interparticle spacing analysis was performed. The edge-to-edge distances between neighboring particles were measured for a statistically relevant number of particles (n = 200). The mean interparticle distance was found to be (43 ± 23) nm. This corresponds to an average interparticle spacing approximately one order of magnitude larger than the mean particle diameter (~ 4.3 nm). In addition, fewer than 4% of the analyzed particles were found to be in direct contact, indicating that the vast majority of particles are spatially separated. While some local variations in particle density are observed,

the overall distribution confirms that the nanoparticles remain largely non-agglomerated after the bubble column and post-treatment steps.

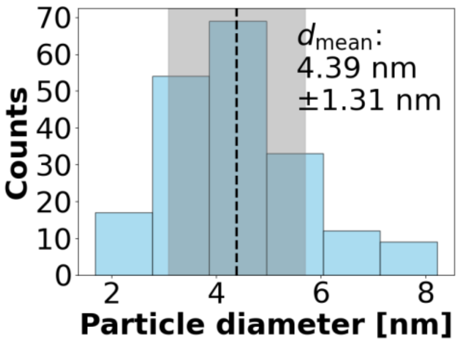

Figure S13: Particle Size Distribution of TEM pictures of Cu NPs supported on VC (8 L/min, 25 °C) after bubble column processing and post-treatment. The distribution is consistent with that obtained prior to the bubble column step.

In addition, the representative TEM images of the Cu nanoparticles supported on Vulcan carbon show that the particle size is preserved during the bubble column and post-treatment steps. The corresponding particle size distribution is shown in Figure S13. The mean particle size and standard deviation are in good agreement with those obtained from TEM analysis of the aerosol-phase nanoparticles prior to the bubble column step (main text, Figure 3), demonstrating that no significant particle growth or agglomeration occurs during processing.

XPS Characterization

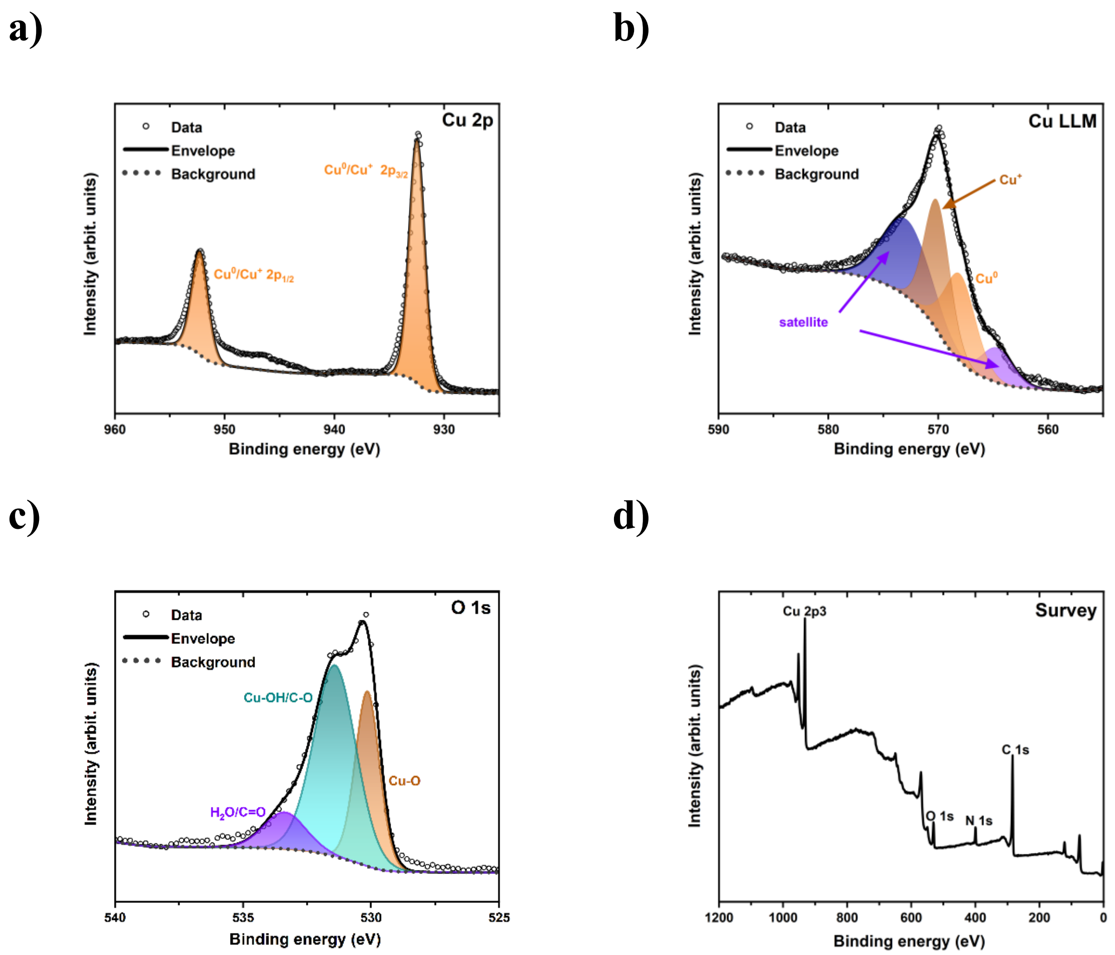

Figure S14: *XPS-spectrum-based determination of the oxidation state of copper in Cu/VC produced by the Spark Ablation/Bubble Column approach (8 L/min, d = 4.3 nm). a) XPS Cu 2p spectra. The peaks at ~ 932.5 eV and ~ 952.3 eV are associated with Cu $2p_{3/2}$ and Cu $2p_{1/2}$ core levels, respectively. b) XPS Cu LMM spectra. The peak associated with metallic Cu^0 is found at ~ 568.0 eV. The peak associated with Cu^+ is found at ~ 570.1 eV. The satellite peaks are located at 564.6 eV and 572.9 eV, respectively. No peak associated with Cu^{2+} can be observed. c) XPS O 1s spectra. d) survey scan.*

X-ray photoelectron spectroscopy (XPS) to determine the oxidation state of Cu was performed with a sample produced by the Spark Ablation/Bubble Column approach (8 L/min, d = 4.3 nm). The deposition time was extended to obtain sufficient signal intensity for reliable XPS analysis. To prepare the sample, an ink was prepared as described in the main text, excluding binder, and drop-cast on a graphite foil, to obtain a sample similar to the electrochemically measured

electrodes. The XPS measurements were carried out on a Kratos Axis Supra instrument. The calibration was performed with the C=C peak at 284.0 eV and the C-C peak at 284.8 eV.

As shown in Figure S14, based on the Cu 2p spectra, the main Cu $2p_{3/2}$ and Cu $2p_{1/2}$ signals were observed at approximately 932.5 eV and 952.3 eV, respectively.[6,7] Since Cu^0 and Cu^+ cannot be unambiguously distinguished by the Cu 2p binding energies alone, the Cu LMM Auger region was additionally analyzed. In the Cu LMM spectrum, contributions at approximately 568.0 eV and 570.1 eV were assigned to Cu^0 and Cu^+, respectively, while no spectral features attributable to Cu^{2+} were observed.[8] Consistent with this, the Cu 2p spectrum did not show the characteristic shake-up satellite structure expected for Cu^{2+} species.[6,7] Quantitative evaluation of the Cu LMM region gave a Cu^0:Cu^+ ratio of 45.3 : 54.7. The O 1s spectrum further showed contributions at approximately 530.1 eV, 531.4 eV, and 533.4 eV, which are consistent with Cu-O, Cu-OH/C-O, and H_2O/C=O species, respectively.[6, 9, 10]

These results show that the as-prepared particles are not dominated by Cu^{2+} oxide species, but instead exhibit a surface composed of metallic Cu and Cu^+ species. These results demonstrate that the surface of the as-prepared electrodes with Cu/VC is only slightly oxidized and composed of metallic Cu and Cu^+ species, which is expected for air-exposed Cu nanoparticles. This brief air exposure during transfer between the glovebox and the XPS is unavoidable and is likely the primary contributor to the observed oxidation.

Reference Electrode Calibration

To ensure reliable and drift-free referencing, particularly important given the sensitivity of peak positions to small shifts in electrode potential, a platinum wire calibration procedure was carried out prior to each measurement session. This was critical to correct for environmental variations (e.g., temperature) that could impact the stability of the three-electrode system. The calibration was conducted in two stages, both using the same three-electrode setup (Pt wire as working electrode, Ag/AgCl reference electrode, and Pt counter electrode), immersed in 0.1 M $HClO_4$ electrolyte.

Electrode preparation:

Prior to immersion, the platinum wire (surface area: 0.196 cm^2) was thoroughly rinsed with ultrapure water and briefly exposed to a flame to remove any residual organic contaminants from its surface.

Stage 1 – Cyclic Voltammetry under Argon Atmosphere

The cell was sparged with high-purity argon gas for 15 minutes to remove dissolved oxygen. A CV was then performed to verify the baseline behavior of the platinum electrode. The characteristic hydrogen adsorption/desorption region and platinum oxide formation/reduction features were used to assess electrode cleanliness and system stability.

CV Parameters:

- Initial potential: −0.250 V vs. Ag/AgCl
- Vertex potential: +1.100 V
- Reverse vertex: −0.250 V
- Final potential: −0.280 V
- Scan rate: 50 mV/s

This scan was typically run for 10 minutes or until stable and reproducible features were observed. Care was taken to minimize noise due to bubbling.

Stage 2 – Calibration in Hydrogen-Saturated Electrolyte

Immediately following the argon scans, the electrolyte was purged with hydrogen gas for 20 minutes to establish equilibrium with the H^+/H_2 redox couple. Under these conditions, the platinum electrode served as a pseudo-reversible hydrogen electrode, allowing precise zero-point calibration against RHE.

CV Parameters:

- Initial potential: −0.195 V vs. Ag/AgCl
- Vertex potentials: −0.200 V and −0.350 V
- Final potential: −0.280 V
- Scan rate: 5 mV/s

Ten cycles were recorded with hydrogen still bubbling through the solution. The zero-potential vs. RHE is defined as the intersection point of the sum of the anodic and cathodic hydrogen currents.

Oxidation Measurements of Cu NPs

After calibration, working electrodes modified with copper nanoparticles were used for the measurement of the oxidation peaks.

Prior to each measurement, a **potential stabilization step** was performed to eliminate capacitive contributions from the electrode interface. A secondary platinum wire ("dummy wire") was connected to the working electrode cable and immersed in the electrolyte at 0.0 V

vs. RHE. The actual working electrode was then slowly introduced into the solution while maintaining the same potential. The platinum wire was subsequently removed. This ensured reproducible initial conditions for each CV experiment.

Cyclic voltammetry (CV) was performed from 0.0 to 1.0 V vs. RHE (or other specific windows such as 0.2–0.8 V), at a scan rate of 1.0 mV/s. This slow scan rate was chosen to approximate reversible electrochemical behavior and to establish a planar diffusion regime. The estimated diffusion layer thickness ($\delta \approx 0.04$ cm) exceeded interparticle spacing, confirming overlapping diffusion zones.

Mass loading was carefully controlled to isolate particle size effects from mass loading influences. Under these conditions, peak potential (E_p) values were extracted and correlated with the nanoparticle radius to study size-dependent oxidation phenomena.

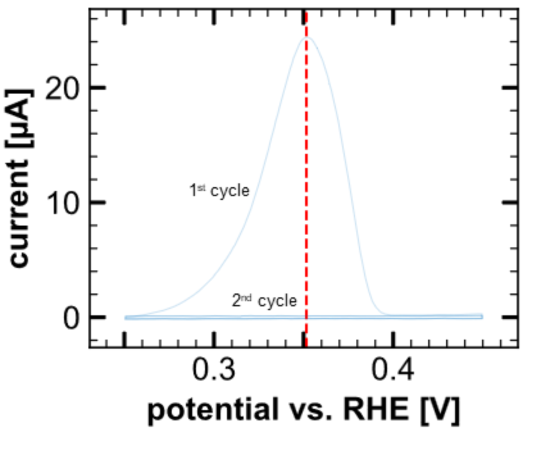

Figure S15: *Typical CV curve (sample: Commercial 25 nm). During the first CV cycle the entire Cu on the electrode is oxidized. The second CV cycle shows no faradaic oxidation peak and only the capacitive current. The contribution from double-layer charging is negligible (<0.1%) for the purpose of mass loading normalization.*

Assumption of Planar Diffusion

The thickness of the diffusion layer can be estimated as $\delta \approx 2(Dt)^{1/2}$. Using $D \approx 1 \times 10^{-5}$ cm^2 s^{-1} and $t \approx 35$ s, the diffusion layer thickness (δ) is calculated to be approximately 370 µm.[11]

The average interparticle distance was estimated assuming a homogeneous distribution of spherical Cu nanoparticles on the carbon support, based on particle size, metal loading, and support surface area. The number of particles was calculated from the total metal mass and the mass of a single particle. The average edge-to-edge distance L was then determined from the particle surface density according to[12]:

$$L = \sqrt{\frac{A}{N}} - d_{NP}$$

where A is the BET surface area of the support and N is the total number of particles. For a Cu loading of 1 wt % and a typical carbon BET surface area of 250 m^2 g^{-1}, the corresponding edge-to-edge distances are ~ 92 nm for particles with a diameter of 4.3 nm and ~ 541 nm for particles with a diameter of 13.8 nm.

To further assess nanoparticle dispersion, an experimental estimate of the interparticle spacing was obtained from TEM analysis for the smallest particle sample (8 L min^{-1}, 25 °C), yielding (43 ± 23) nm (see SI Section: *TEM Analysis after Bubble Column Collection/Post-Treatment*).

Since the diffusion layer thickness is several orders of magnitude larger than the interparticle spacing, substantial overlap of diffusion fields is expected, supporting the applicability of a planar diffusion description under the employed experimental conditions.

Effect of Mass Loading on the Oxidation Peak Potential

Figure S16a shows representative CVs of Cu nanoparticles (sample: commercial 25 nm) recorded at different mass loadings. The mass loading was adjusted by varying the volume of ink drop-cast onto the electrode. The corresponding measured data are summarized in Table S1. Figure S16b presents the oxidation peak potential (E_p) plotted against the logarithm of the charge under the peak ($\ln Q$). The charge under the oxidation peak is directly proportional to the mass loading according to Faraday's law. As expected, E_p and $\ln Q$ exhibit a linear correlation. Within the narrow mass loading range used for the size-dependent measurements, the influence of mass loading on E_p is minimal and described in the main text.

Table S1: Mass loading Q and respective oxidation peak potentials (E_p) of commercial 25 nm Cu nanoparticles

Charge under the Peak Q [mC]	ln(Q)	Peak Potential [mV] vs. RHE
2.50	-5.99	354.7
2.14	-6.15	352.5
1.74	-6.35	349.6
1.30	-6.65	351.6
1.10	-6.81	343.6

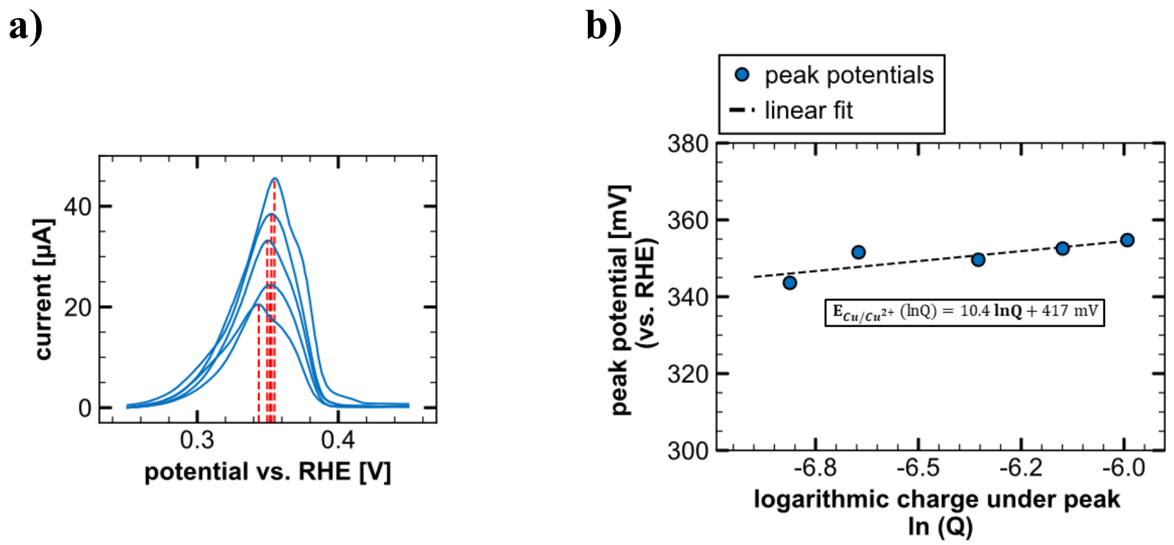

Figure S16: Mass loading dependency of oxidation peak potentials (E_p). a) CVs of Cu nanoparticles (sample: commercial 25 nm) recorded at different mass loadings. b) Oxidation peak potential (E_p) plotted against the logarithm of the charge under the peak ($\ln Q$). The expected linear correlation between E_p and $\ln(Q)$ can be observed.

Effect of Loading (w.-% Cu/VC) on the Oxidation Peak Potential

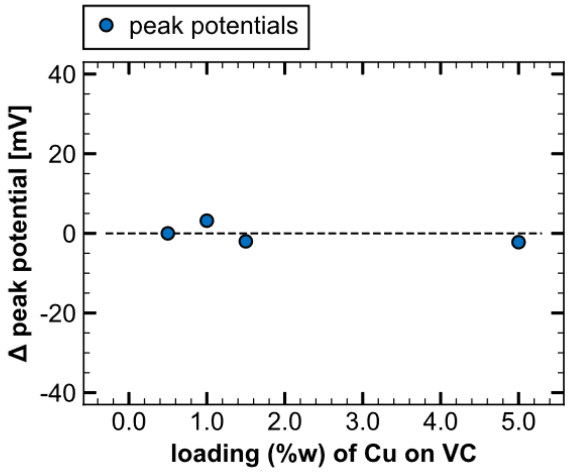

Figure S17: *Loading dependency of oxidation peak potentials (E_p) for Cu nanoparticles supported on VC (sample: 4.3 nm, produced via Spark Ablation/Bubble column approach). The Oxidation peak potential (E_p) is plotted against the loading (w.-% Cu on VC). No correlation between E_p and the loading (w.-% Cu on VC) can be observed.*

Figure S17 presents the peak potential variations of Cu nanoparticles (sample: 4.3 nm, produced via Spark Ablation) measured at different loadings (w.-% Cu on VC). The loading was tuned by adjusting the deposition time, while the total mass loading, reflected by the charge under the oxidation peak, was kept approximately constant by varying the drop-cast volume. As illustrated in Figure S17, no correlation is observed between the Cu loading (w.-% on VC) and the peak potential.

Effect of Nanoparticle Size Dispersity on Oxidation Peak Width

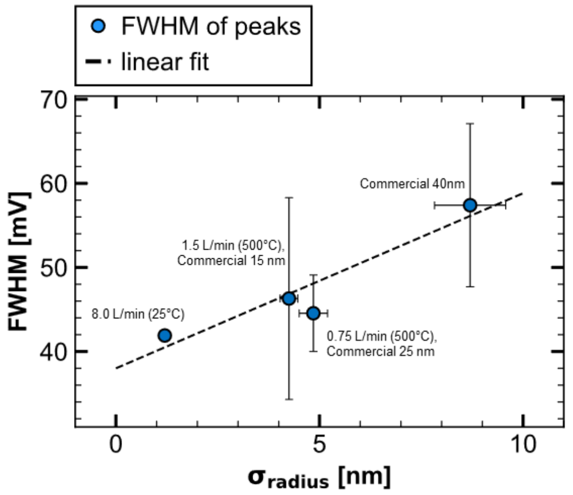

Figure S18: *Effect of nanoparticle size dispersity (δ_{radius}) on oxidation peak width (FWHM). A linear correlation can be observed.*

To evaluate the effect of nanoparticle size dispersity on the oxidation peak width, the FWHM obtained from electrochemical measurements (see Table 1, main text) was plotted as a function of the nanoparticle size dispersity (δ_{radius}) determined from SEM/TEM analysis (Figure S18). Samples with very similar dispersity values (δ_{radius}) and ones that agreed within their respective uncertainties ($\Delta\delta_{\text{radius}}$) were grouped. A linear correlation between δ_{radius} and the FWHM is observed ($R^2 = 0.89$), following the relation: $\text{FWHM}_{\text{Cu, ox}} = 2.08\, {}^{mV}/_{nm} \cdot \delta_{\text{radius}} + 37.6\ \text{mV}$. Owing to the limited number of data points, the observed relationship should be interpreted as a preliminary trend rather than a conclusive correlation.

Model by Plieth (Fitting)

a)

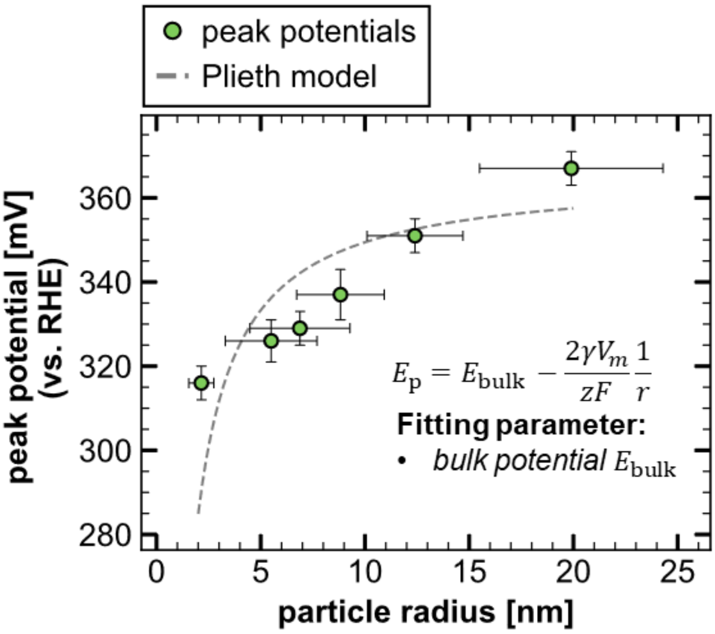

b)

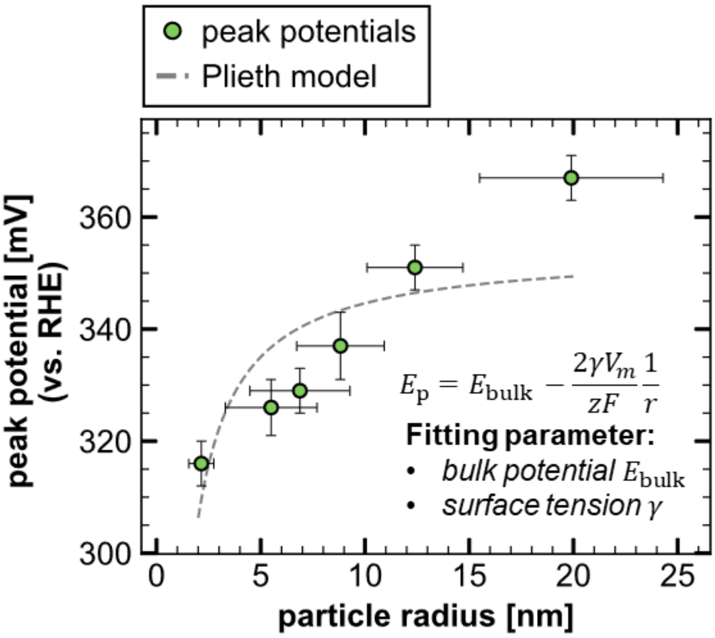

Figure S19: Theoretical Model proposed by Plieth.[13] Fitting the thermodynamically derived equation of Plieth to the experimental data of Cu NPs using a) only the bulk potential as fitting parameter (E_{bulk} = 365.6 mV). b) the bulk potential and the surface tension as fitting parameters (E_{bulk} = 354.2 mV, γ_{fit} = 1299.6 erg/cm^2).

Linear Fit for Copper and Silver Nanoparticles (data from Ivanova et al.)

Table S2: Oxidation peak potentials from Ivanova et al.[14] for Ag NPs in the same particle size range as the Cu NPs in this study (diameter ≤ 40 nm)

Average diameter SEM/AFM [nm]	Peak Potential [mV] vs. RHE
10.4 (± 2.4)	275 (± 25)
16.8 (± 4.4)	291 (± 11)
21.0 (± 3.8)	318 (± 6)
25.8 (± 4.1)	340 (± 4)
29.4 (± 6.2)	354 (± 7)
36.5 (± 8.3)	371 (± 10)

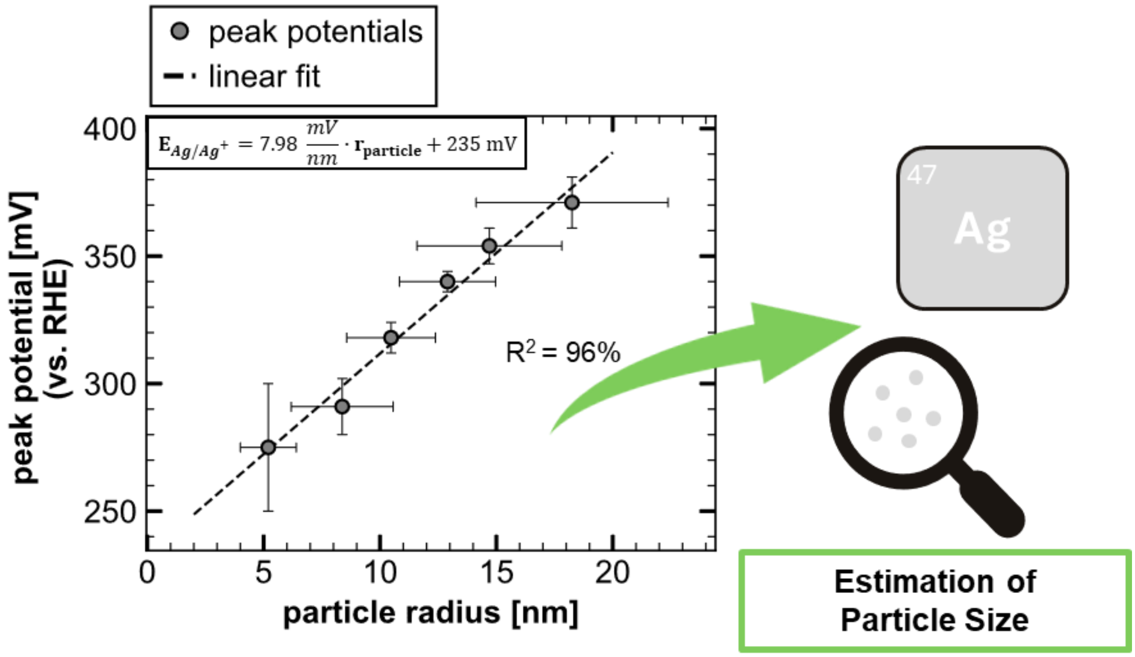

Figure S20: Re-evaluation of the oxidation peak potentials from Ivanova et al. for Ag NPs in the same particle size range as the Cu NPs in this study (diameter ≤ 40 nm). A linear correlation (R^2 = 0.96) between particle radius and peak potential is observed. The average E_p values shift positive with increasing Cu NP size (radius). The established linear correlation can be used to estimate the particle size of Ag NPs by direct electrochemical measurements.

Table S3: Linear regression parameters describing the dependence of oxidation potential on particle radius. Weighting considering the errors was applied (instrumental weighting). Parameter uncertainties are reported as standard errors (SE) and 95% confidence intervals (CI) calculated from the weighted least-squares regression (OriginPro 2025).

**Parameter**	**value**	**standard error (SE)**	**95% CI (lower)**	**95% CI (upper)**
$E_{\mathrm{Cu,\,ox}} = 2.96\ ^{mV}/_{nm} \cdot r_{\mathrm{particle}} + 310$ **mV** ($R^2 = 0.99$)				
Intercept Cu NPs [mV]	310.21	± 2.04	304.54	315.88
Slope Cu NPs [mV/nm]	2.96	± 0.18	2.46	3.46
$E_{\mathrm{Ag,\,ox}} = 7.98\ ^{mV}/_{nm} \cdot r_{\mathrm{particle}} + 235$ **mV** ($R^2 = 0.96$)				
Intercept Ag NPs [mV]	234.66	± 10.95	204.26	265.05
Slope Ag NPs [mV/nm]	7.98	± 0.85	5.63	10.33

References

1. Lu, J.; Guo, J.; Song, S.; Yu, G.; Liu, H.; Yang, X.; Lu, Z. Preparation of Ag Nanoparticles by Spark Ablation in Gas as Catalysts for Electrocatalytic Hydrogen Production. RSC Adv. 2020, 10 (63), 38583–38587. https://doi.org/10.1039/d0ra06682f.

2. Tabrizi, N. S.; Ullmann, M.; Vons, V. A.; Lafont, U.; Schmidt-Ott, A. Generation of Nanoparticles by Spark Discharge. J Nanopart Res 2008, 11 (2), 315–332. https://doi.org/10.1007/s11051-008-9407-y.

3. Spark Ablation; Schmidt-Ott, A., Ed.; Jenny Stanford Publishing, 2019. https://doi.org/10.1201/9780367817091.

4. Ternero, P.; Preger, C.; Eriksson, A. C.; Rissler, J.; Hübner, J.-M.; Messing, M. E. In-Flight Tuning of Au–Sn Nanoparticle Properties. Langmuir, 2024, 40 (31), 16393–16399. https://doi.org/10.1021/acs.langmuir.4c01656.

5. Lamb, D.; Hobbs, P. V. Aerosol Particles from Biomass Burning. J. Aerosol Sci. 1980, 11 (5), 491–505. https://doi.org/10.1016/S0021-8502(05)80005-9

6. Biesinger, M. C. Advanced Analysis of Copper X-ray Photoelectron Spectra. Surf. Interface Analysis, 2017, 49 (13), 1325–1334. https://doi.org/10.1002/sia.6239.

7. Moretti, G.; Fierro, G.; Lo Jacono, M.; Porta, P. Characterization of CuO–ZnO Catalysts by X-ray Photoelectron Spectroscopy: Precursors, Calcined and Reduced Samples. Surf. Interface Analysis, 1989, 14 (6–7), 325–336. https://doi.org/10.1002/sia.740140609.

8. Aria, A. I.; Kidambi, P. R.; Weatherup, R. S.; Xiao, L.; Williams, J. A.; Hofmann, S. Time Evolution of the Wettability of Supported Graphene under Ambient Air Exposure. J. Phys. Chem. C, 2016, 120 (4), 2215–2224. https://doi.org/10.1021/acs.jpcc.5b10492.

9. Smith, M.; Scudiero, L.; Espinal, J.; McEwen, J.-S.; Garcia-Perez, M. Improving the Deconvolution and Interpretation of XPS Spectra from Chars by Ab Initio Calculations. Carbon, 2016, 110, 155–171. https://doi.org/10.1016/j.carbon.2016.09.012.

10. Pirug, G.; Ritke, C.; Bonzel, H. P. Adsorption of H2O on Ru(001). Surface Science, 1991, 241 (3), 289–301. https://doi.org/10.1016/0039-6028(91)90089-b.

11. Marcinkowsky, A. E.; Phillips, H. O. Diffusion Studies. Part II. Tracer Diffusion Coefficients of Copper(II) in HCl and HClO4 at 25 °C. J. Chem. Soc. A, 1971, 0 (0), 101–103. https://doi.org/10.1039/j19710000101.

12. Speder, J.; Altmann, L.; Bäumer, M.; Kirkensgaard, J. J. K.; Mortensen, K.; Arenz, M. The Particle Proximity Effect: From Model to High Surface Area Fuel Cell Catalysts. RSC Adv., 2014, 4 (29), 14971. https://doi.org/10.1039/c4ra00261j.

13. Plieth, W. J. Electrochemical Properties of Small Clusters of Metal Atoms and Their Role in the Surface Enhanced Raman Scattering. J. Phys. Chem., 1982, 86 (16), 3166–3170. https://doi.org/10.1021/j100213a020.

14. Ivanova, O. S.; Zamborini, F. P. Size-Dependent Electrochemical Oxidation of Silver Nanoparticles. J. Am. Chem. Soc. 2009, 132 (1), 70–72. https://doi.org/10.1021/ja908780g.
